# Neonatal Exposure to Antibiotics Triggers Obesity in Adulthood by Permanent Microbiota Dysbiosis and Reduction of Adipose Regulatory T Cells

**DOI:** 10.1002/dni2.70003

**Published:** 2026-09-16

**Authors:** Peter Zanvit, Junji Xu, Wenjing Yang, Nancy Guo, Tianming Yu, Xue Jiao, Michaela Prochazkova, Wenwen Jin, Na Liu, Sang-A Park, Cheryl Chia, Daxesh P. Patel, Dunfang Zhang, Nathan Goldberg, Alexander Cain, Frank J. Gonzalez, Yasmine Belkaid, Yingzi Cong, WanJun Chen

**Affiliations:** 1 Mucosal Immunology Section, National Institute of Dental and Craniofacial Research (NIDCR), National Institutes of Health, Bethesda, Maryland, USA; 2 Division of Gastroenterology and Hepatology, Department of Medicine, Northwestern University, Chicago, Illinois, USA; 3 Department of Microbiology and Immunology, University of Texas Medical Branch, Galveston, Texas, USA; 4 Department of Transfusion Medicine, Center for Cellular Engineering, NIH, Bethesda, Maryland, USA; 5 Center for Cancer Research, NCI, NIH, Bethesda, Maryland, USA; 6 Metaorganism Immunity Section, Laboratory of Immune System Biology, National Institute of Allergy and Infectious Diseases, Bethesda, Maryland, USA; 7 NIAID Microbiome Program, National Institute of Allergy and Infectious Diseases, Bethesda, Maryland, USA

**Keywords:** inflammation, intestinal permeability, microbiota dysbiosis, neonatal age, obesity, ST2, TGFβ signaling, Treg

## Abstract

Neonatal dysbiosis of the gut microbiota represents a critical environmental factor promoting obesity, but the underlying immunological mechanisms remain unknown. We show here that neonatal treatment of mice with broad-spectrum antibiotics for the first 3 weeks of life (NeoATB) resulted in the development of obesity and metabolic abnormalities in adulthood. The NeoATB mice exhibited a permanent dysbiosis of gut microbiota, decreased CD4^+^Foxp3^+^ regulatory T cells (Tregs), and increased proinflammatory Th1 cells in visceral adipose tissue (VAT). Mechanistically, neonatal antibiotic treatment resulted in increased intestinal permeability that allowed bacterial translocation into the VAT and liver and an increase in systemic LPS levels. Consequently, VAT CD11c^+^MHCII^+^ cells were activated via the TLR4 pathway to increase IL-12-triggered Th1-inflammation. Moreover, the decrease in VAT Tregs in NeoATB mice was attributed to reduced infiltration of Tregs from the periphery, decreased expansion of adipose IL-33-mediated ST2^+^ Tregs, and reduced conversion of local Tregs from VAT CD4^+^CD25^−^ Foxp3^−^ T cells. Thus, we have revealed a previously unrecognized immunological link between neonatal microbiota dysbiosis and the development of obesity.

## 1 | Introduction

Over the past few years, the field of immunology has been significantly revolutionized by an understanding of the fundamental role of microbiota in the induction, education, and function of the mammalian immune system [1]. In humans, the number of bacteria in the body is actually of the same order as the number of human nucleated cells (3.8 × 10^13^–3 × 10^13^), and their total mass is about 0.2 kg [2]. Acquisition of microbiota begins at birth, which represents the first major exposure of the newborn to microbes, and it may depend on the type of delivery [3, 4]. In addition, antibiotic use during the neonatal age can have striking effects on the early microbiota composition and metabolism [5, 6]. The microbiota composition of an adult may, therefore, be influenced greatly by the history of exposure to microbes and environmental factors early in life. In the last decades, there has been a surge in the incidence of autoimmune diseases, obesity, and metabolic syndrome globally. The gut microbiota is altered in obesity, and recent studies have shown that the gut microbiota is considered a critical environmental factor that promotes adiposity and contributes to obesity [7, 8]. Obesity is accompanied by chronic low-grade inflammation of adipose tissue, which promotes insulin resistance and type 2 diabetes. Among different cell types in adipose tissue, CD4^+^CD25^+^ Foxp3^+^ regulatory T cells (Treg) were identified as the key players in regulating adipose tissue inflammation [9]. However, the mechanisms by which the early dysbiosis of microbiota during the neonatal age drives dysregulated immune responses in the adipose tissue and triggers the development of obesity remain unknown.

We show here that the use of antibiotics at neonatal age in mice (NeoATB) promotes the development of obesity and metabolic abnormalities, including glucose intolerance and insulin resistance later in life. We demonstrated that adult NeoATB obese mice displayed a long-lasting microbiota dysbiosis in the gut, and significantly decreased immunoregulatory CD4^+^CD25^+^ Foxp3^+^ Tregs and consequently increased Th1-type inflammation in the VAT. We further elucidated that neonatal antibiotic treatment-mediated microbiota dysbiosis resulted in deficient production of gut short-chain fatty acids (SCFAs) that, together with decreased IL-22-producing T cells, contributed to the increased intestinal permeability. Increased intestinal permeability led to systemic translocation of bacteria in the VAT and liver and increased serum levels of LPS to promote Th1-type inflammation by triggering IL-12 production by CD11c^+^ MHCII^+^ cells in VAT. Furthermore, we also revealed that the decrease in VAT Tregs in NeoATB mice was attributed to the deficiency of local conversion from CD4^+^ Foxp3^−^ T cells and reduction of CD25^+^ST2^+^Foxp3^+^ Treg cell proliferation in the adipose tissue as well as reduced infiltration from the peripheral Treg pool. These findings collectively point to a previously unrecognized link between neonatal dysbiosis of the gut microbiome and obesity and abnormal metabolism in adulthood.

## 2 | Results

### 2.1 | Antibiotics in Neonatal Life Cause Obesity in Adulthood

**FIGURE 1 |:**
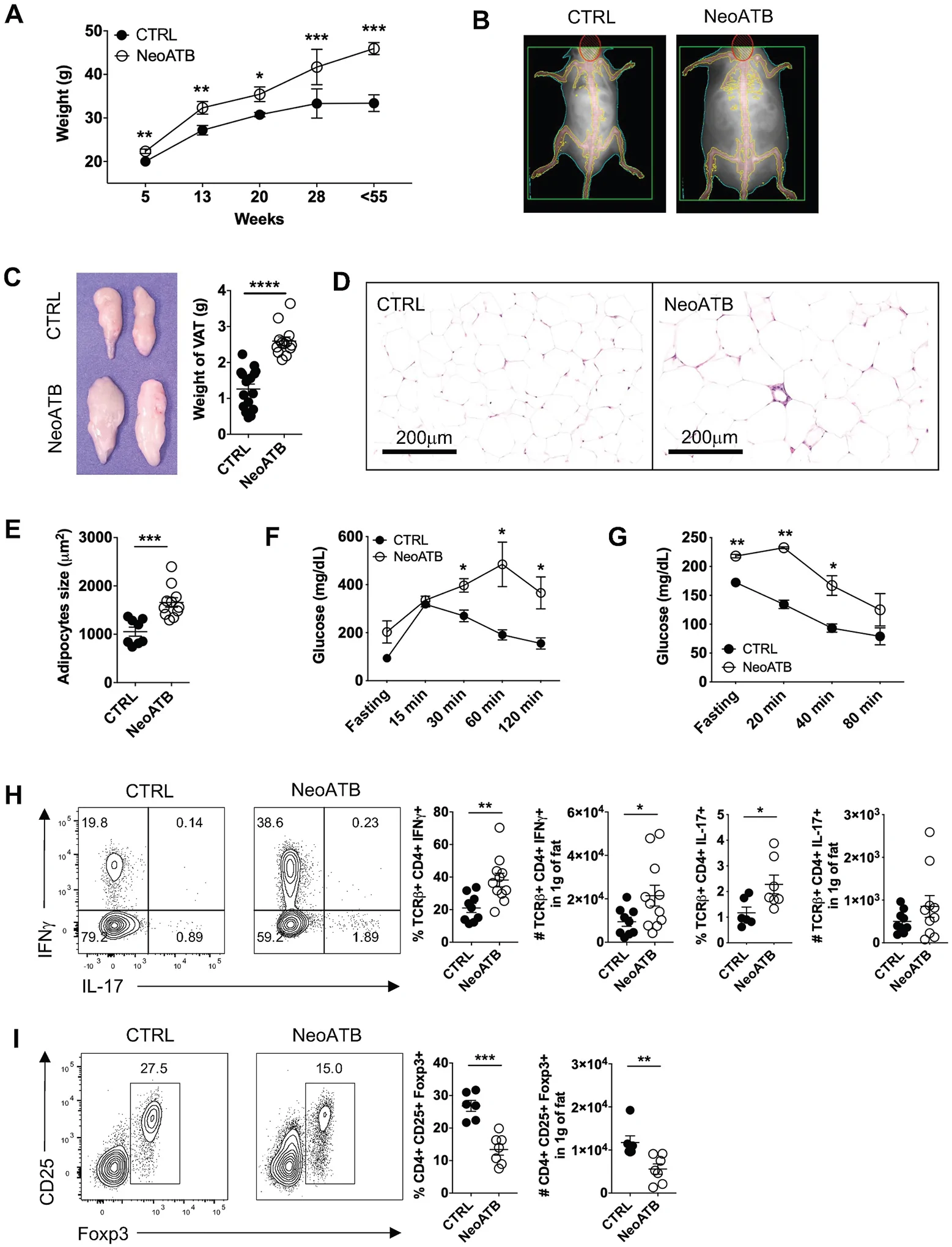
Antibiotic treatment in neonatal age exacerbates development of obesity in adulthood. Mice were treated with antibiotics vancomycin + polymyxin B (NeoATB) for the first 3 weeks of life or left untreated (Control; CTRL) and then housed in a normal environment. (A) The summarizing dot plot shows body weight of control (CTRL) and NeoATB mice at different ages. (B) Dual energy X-ray absorptiometry (DEXA) of adult control or NeoATB obese mice. (C) Photograph of visceral adipose tissue (VAT) of ~30-week-old CTRL and NeoATB male mice. The summarizing dot plot shows the weight of VAT of CTRL and NeoATB mice. (D) H&E staining of visceral adipose tissue of ~30-week-old CTRL and NeoATB mice. Scale bar = 200 μm. (E) The area of adipocytes of control and NeoATB mice was determined by Adiposoft software (http://imagej.net/Adiposoft). (F) Glucose tolerance test of adult ~30-week-old CTRL and NeoATB mice. (G) Insulin tolerance test of adult ~30 weeks of CTRL and NeoATB mice. (H) Representative FACS plots showing the frequency of CD4^+^IFNγ^+^ (Th1) or CD4^+^IL-17^+^ (Th17) cells in adipose tissue of ~30-week-old CTRL and NeoATB mice. Dot plots showing summarized frequencies and total numbers of adipose Th1 and Th17 cells in CTRL and NeoATB mice. (I) Representative FACS plots showing the frequency of CD4^+^CD25^+^Foxp3^+^ (Treg) cells in adipose tissue of ~30-week-old CTRL and NeoATB mice. Dot plots show summarized frequencies and total numbers of adipose Treg cells in CTRL and NeoATB mice. Data is pooled from 2 to 3 independent experiments. Significance was determined using a two-tailed Student *t*-test (*p < 0.05, **p < 0.01, ***p < 0.001).

We have previously demonstrated that antibiotics in neonatal life (first 3 weeks; NeoATB; see immunization scheme in Supporting Information S1: Figure S1A) increased murine susceptibility to experimental psoriasis [10]. Surprisingly, when NeoATB mice reached ~30 weeks of age, they displayed significantly increased body weight compared to untreated mice (Figure 1A,B). NeoATB mice also had significantly increased white adipose tissue (VAT) weight and larger adipocyte size compared to control mice (Figure 1C–E). The obesity in adult NeoATB mice correlated with increased fasting glucose, glucose intolerance, and insulin resistance (Figure 1F,G).

In line with a previous report linking Th1 responses to metabolic syndrome [11], obese NeoATB mice harbored increased frequency and absolute number of CD4^+^IFNγ^+^ (Th1) cells in VAT (Figure 1H). Moreover, the frequency and total number of TCRγδ^+^IFNγ^+^ cells were significantly increased in the VAT of NeoATB mice (Supporting Information S1: Figure S1B). We also found an increase in frequency but not in the total number of Th17 cells in the VAT of NeoATB mice compared to controls (Figure 1H). Intriguingly, the frequency of TCRγδ^+^IL-17^+^ cells was significantly decreased, although the absolute number was unaltered (Supporting Information S1: Figure S1B). In contrast, Th2 cytokines IL-4, IL-5, and IL-13 were not altered between control and NeoATB mice (Supporting Information S1: Figure S1C). In addition to adaptive immunity, an increase in the number and function of macrophages in adipose tissue was linked with obesity-associated inflammation and dysregulation of metabolism [12, 13]. Consistently, we observed an increased frequency and total number of CD11b^+^F4/80^+^ myeloid cells in the VAT of NeoATB mice (Supporting Information S1: Figure S1D).

Adipose Treg cells were shown to play a crucial role in regulating VAT inflammation and, consequently, in controlling weight gain and metabolism [9]. We next determined that NeoATB mice exhibited significantly decreased frequency and total number of CD4^+^Foxp3^+^ Treg cells (Figure 1I) as well as the ST2^+^ Treg subset in VAT (Supporting Information S1: Figure S1E). Moreover, innate lymphoid cells type 2 (ILC2, CD45^+^Lin^−^ SCA1^−^ ST2^+^) were also significantly reduced in the VAT of NeoATB mice (Supporting Information S1: Figure S1F), which is consistent with the notion that ILC2 regulates obesity development both in humans and mice [14]. These data support the idea that antibiotic exposure in neonatal life promotes the development of obesity in adulthood, which was accompanied by decreased Treg cells and increased Th1 inflammation in the VAT.

### 2.2 | Altered Gut Microbiota in NeoATB Mice

**FIGURE 2 |:**
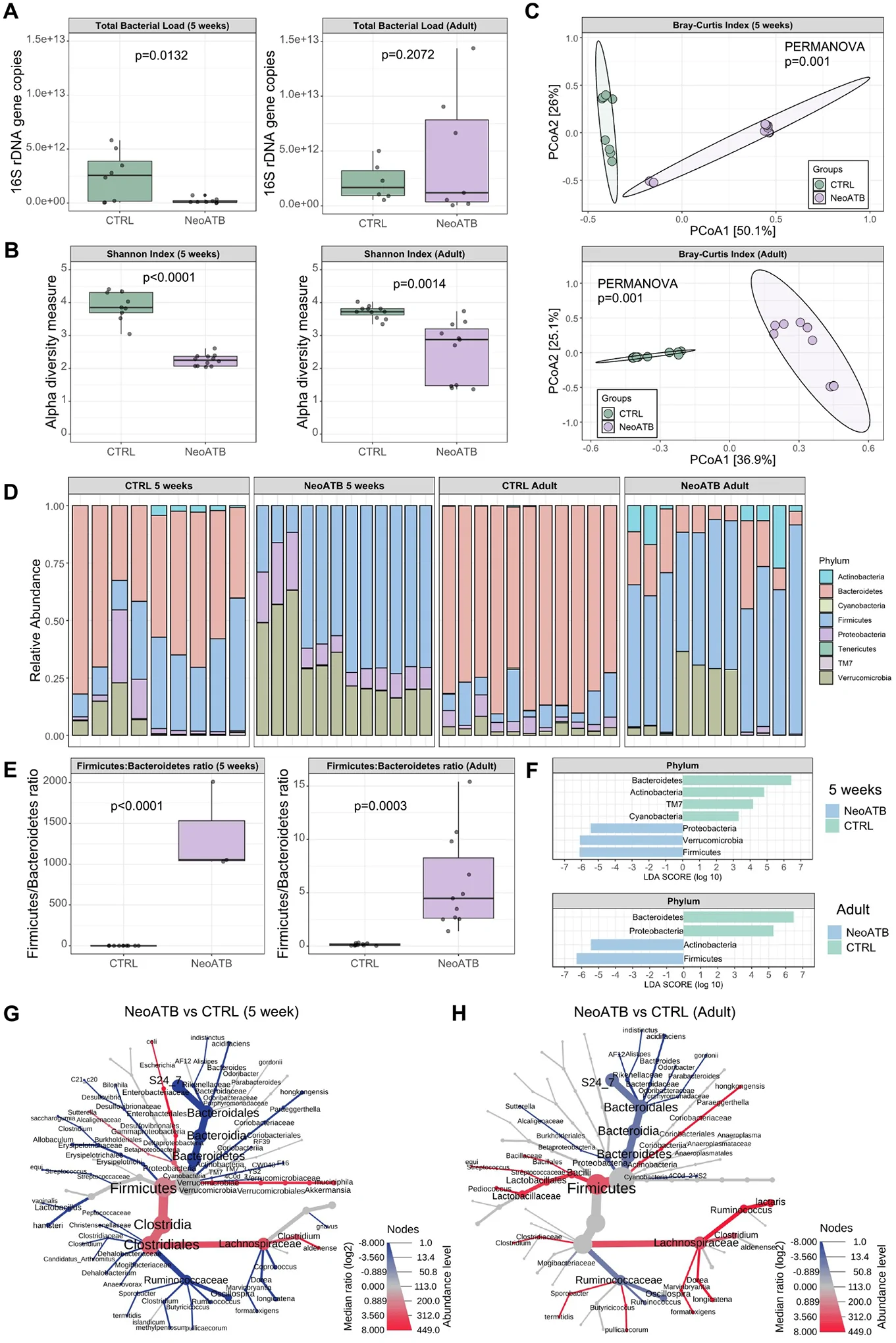
Dysregulated microbiota profile in adult NeoATB mice. (A) Total bacterial load was determined as the number of 16S rDNA gene copies using qPCR in the feces of CTRL and NeoATB mice. (B) Alpha diversity measure (Shannon Index) in feces of 5-week-old and adult (30 weeks old) CTRL and NeoATB mice. (C) Beta diversity measure (Bray–Curtis Index) in feces of 5-week-old and adult CTRL and NeoATB mice. (D) Phylum-level taxa abundance in feces of 5-week-old and adult CTRL and NeoATB mice. (E) *Firmicutes* to *Bacteroidetes* ratio in 5-week-old and adult CTRL and NeoATB mice. (F) Linear discriminant analysis showing significantly different taxa on the phylum level in feces of 5-week-old and adult mice determined by LEfSe analysis (*p* < 0.05, FDR = 0.05, LDA = 3) (G) Heat tree analysis showing significantly different taxa detected in the feces of 5-week-old NeoATB compared to age-matched CTRL mice. (H) Heat tree analysis showing significantly different taxa detected in the feces of adult NeoATB compared to age-matched CTRL mice. Heat tree analysis uses the hierarchical structure of taxonomic classifications to quantitatively (based on median abundance) and statistically (based on the Wilcoxon rank sum test) depict significant taxon differences among communities.

Compelling evidence has shown that altered microbiota composition in early life contributes to obesity [15]. Next, we compared the fecal microbiota profile in young (5-weeks-old) and adult NeoATB (30-week-old) mice with age-matched control mice. Total bacterial load was significantly reduced in 5-week-old NeoATB mice compared to matched control mice but not in adult NeoATB mice (Figure 2A). Five-week-old NeoATB mice had increased size and weight of cecum compared to age-matched controls, supporting the view that accumulation of dietary fiber components could result from reduced bacterial load (Supporting Information S1: Figure S3A). Alpha-diversity analysis (Shannon index) revealed significantly reduced microbiota diversity in both 5-week-old and adult NeoATB mice compared to age-matched control mice (Figure 2B). On the other hand, the beta diversity (PCoA, Bray-Curtis index) analysis showed significant differences in clustering between microbiota in the 5-week-old and adult control and NeoATB mice (Figure 2C). At the phylum level, the phyla *Tenericutes* and *Verrucomicrobia* (in feces of 5-week-old mice) or *Tenericutes*, *Verrucomicrobia*, and *Firmicutes* (in feces of adult mice) were significantly more abundant (LDA = 3, *p* < 0.05, FDR *p* < 0.05) when compared with age-matched controls (Figure 2D,F, Supporting Information S1: Figure S2A,B). On the other hand, the phyla *Bacteroidetes, Cyanobacteria, and TM7* (in the feces of 5-week-old control mice) or the phylum *Bacteroidetes* (in the feces of adult control mice) were significantly more abundant (LDA = 3, *p* < 0.05, FDR *p* < 0.05) when compared with age-matched NeoATB mice (Figure 2D,F, Supporting Information S1: Figure S2A,B). A significantly increased ratio of the phylum *Firmicutes* to *Bacteroidetes* was detected in the feces of both 5-week-old and adult NeoATB mice compared to age-matched controls (Figure 2E). Neonatal antibiotic treatment decreased levels of microbial-derived metabolites—short-chain fatty acids (SCFA), mostly butyrate and propionate in 5-week-old NeoATB mice (Supporting Information S1: Figure S3B). In adult mice, the levels of acetate and butyrate were no longer altered between control and NeoATB mice, although NeoATB mice showed significantly increased levels of propionate (Supporting Information S1: Figure S3C).

Further, we utilized Heat tree analysis—a powerful method that allowed us to visually compare abundance between two groups (NeoATB vs. CTRL) at different taxonomic levels. In the feces of NeoATB mice aged 5 weeks, we detected a significant increase in the abundance of the following microbial families: *Lachnospiraceae* (*p* = 0.01838533), *Enterobacteriaceae* (*p* = 0.00014065), *Verrucomicrobiaceae* (*p* = 0.00020413), *Aneroplasmataceae* (*p* = 0.03014007), and *Alcaligenaceae* (*p* = 2.72e-05). Previous reports have suggested that the families *Lachnospiraceae* and *Enterobacteriaceae* are associated with the development of obesity [16–18]. The family *Lachnospiraceae* (*p* = 0.00027501) remained significantly more abundant in the feces of adult NeoATB mice compared to control mice. Moreover, we detected significantly increased abundance of the families Bacillaceae (*p* = 0.04308641), Lactobacillaceae (*p* = 8.11e-05), Clostridiaceae (*p* = 0.00711958), Coriobacteriaceae (*p* = 0.00255111), and Streptococcaceae (*p* = 0.00654733) in the feces of adult obese NeoATB mice compared to age-matched control mice.

Next, two independent fecal microbiota transfer (FMT) experiments were performed to answer the question of whether microbiota composition in NeoATB mice plays a role in obesity development in these mice. In these experiments, 5-week-old age-matched male germ-free mice were used. Germ-free mice were orally given fecal suspension (collected from 5-week-old CTRL or NeoATB mice) 2 times at weeks 0 and 1. Mice were then sacrificed 8 weeks later. After 8 weeks, germ-free mice implanted with fecal microbiota that were collected from NeoATB mice developed an obesity phenotype. In these mice, we observed significantly increased body weight as well as significantly increased weight of VAT (Supporting Information S1: Figure S4A,B). Importantly, the FITC-dextran assay confirmed increased intestinal permeability in germ-free mice implanted with fecal suspension from NeoATB mice (Supporting Information S1: Figure S4C). On the other hand, germ-free mice implanted with fecal suspension from control mice did not develop obesity nor increased intestinal permeability. In the adipose tissue of germ-free mice implanted with fecal microbiota suspension from NeoATB mice, we detected a significantly increased total number of CD4^+^IFNγ^+^ cells as well as a higher frequency of CD8^+^IFNγ^+^ cells (Supporting Information S1: Figure S4D,E). Intriguingly, we did not observe a significant difference in frequency and total number of CD4^+^Foxp3^+^D25^+^ regulatory T cells as well as CD11b^+^F4/80^+^ myeloid cells in the fat of germ-free mice implanted with fecal suspension of NeoATB mice (Supporting Information S1: Figure S4F,G).

To address whether obesity in NeoATB mice could be induced by antibiotic treatment per se, 6-week-old germ-free male mice were treated with antibiotics (Vancomycin and Polymyxin B) for 3 weeks and then switched to regular water for another 5 weeks. Our data indicate that antibiotic treatment did not affect germ-free mice’s body weight or VAT weight (Supporting Information S1: Figure S5A,B). Importantly, antibiotic treatment of germ-free mice had no effect on intestinal permeability (Supporting Information S1: Figure S5C) nor the induction of inflammation in the VAT. No difference in frequency or total number of CD4^+^IFNγ^+^, CD4^+^IL-17^+^, CD8^+^IFNγ^+^, CD4^+^Foxp3^+,^ as well as CD11b^+^F4/80^+^ cells was observed (Supporting Information S1: Figure S5D–H). Overall, the data indicate that gut microbiota composition in NeoATB mice is responsible for increased intestinal permeability and the development of obesity. Moreover, our data revealed a long-lasting effect of neonatal antibiotic treatment on the gut microbiota, a phenomenon that may contribute to long-term immune and metabolic alterations.

### 2.3 | Early Onset of Fat Inflammation in NeoATB Mice

**FIGURE 3 |:**
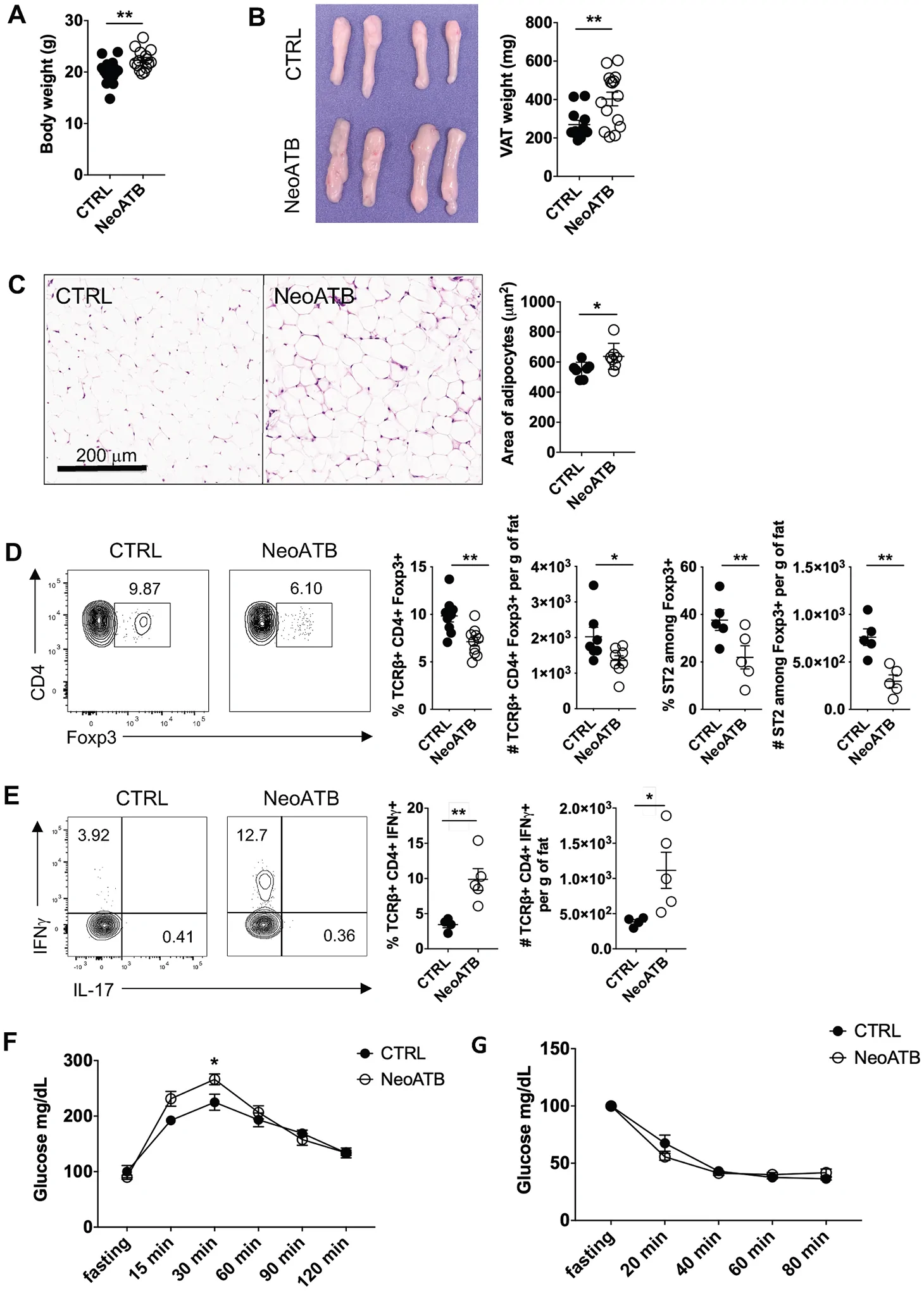
Early onset of obesity in 5-week-old NeoATB mice. (A) The summarizing dot plot shows the body weight of 5-week-old control (CTRL) and NeoATB mice. (B) Photograph of visceral adipose tissue (VAT) of 5-week-old CTRL and NeoATB male mice. The summarizing dot plot shows the weight of VAT of 5-week-old CTRL and NeoATB mice. (C) H&E staining of visceral adipose tissue of 5-week-old CTRL and NeoATB mice. Scale bar = 200 μm. The area of adipocytes of control and NeoATB mice was determined by Adiposoft software (http://imagej.net/Adiposoft). (D) Representative FACS plots showing the frequency of TCRβ^+^CD4^+^Foxp3^+^ (Treg) cells in adipose tissue of 5-week-old CTRL and NeoATB mice. Dot plots show summarized frequencies and total numbers of adipose TCRβ^+^CD4^+^Foxp3^+^ cells or the total number of ST2^+^ cells among Foxp3^+^ Treg cells in CTRL and NeoATB mice. (E) Representative FACS plots showing the frequency of CD4^+^IFNγ^+^ (Th1) cells in adipose tissue of 5-week-old CTRL and NeoATB mice. Dot plots show summarized frequencies and total numbers of adipose Th1 cells of 5-week-old CTRL and NeoATB mice. (F) Glucose tolerance test of adult 5-week-old CTRL and NeoATB mice. (G) Insulin tolerance test of adult 5-week-old CTRL and NeoATB mice. Data are pooled from 2 to 3 independent experiments. Significance was determined using a two-tailed Student *t*-test (*p < 0.05, **p < 0.01, ***p < 0.001).

Next, we investigated the underlying immunological mechanisms responsible for neonatal antibiotic treatment-mediated obesity in adult mice. To address this issue, mice that had been treated with antibiotics for the first 3 weeks of life and fed with normal chow (Supporting Information S1: Figure S1A) were examined at 5 weeks of age. Age 5 weeks was selected because mice younger than 5 weeks had very limited adipose tissue depots. Surprisingly, NeoATB mice started to display early onset of obesity at 5 weeks of age as indicated by body weight (Figure 3A) and increased weight of VAT (Figure 3B). Histological analysis of the VAT revealed an increased size of adipocytes in NeoATB mice compared to control mice (Figure 3C). Consistent with a previous report [9], 5-week-old mice had a relatively low frequency (~10%) of CD4^+^Foxp3^+^ cells in the VAT. However, neonatal antibiotic treatment significantly decreased the frequency and the total number of Treg cells in adipose tissue of 5-week-old NeoATB mice (Figure 3D). Importantly, the numbers of ST2^+^Foxp3^+^ Treg cells that are recognized as key effector Tregs in suppressing inflammation in adipose tissue [9] were also significantly decreased in VAT of 5-week-old NeoATB mice compared to control mice (Figure 3D). In contrast, the frequency and total number of Th1 (CD4^+^IFNγ^+^) (Figure 3E) as well as CD3^+^IFNγ^+^ or CD3^+^IFNγ^+^TNF^+^ cells were significantly higher in the VAT of NeoATB mice (Supporting Information S1: Figure S3A,B). In addition, we observed an increased total number of CD11b^+^F4/80^+^ and proinflammatory CD11b^+^F4/80^+^IFNγ^+^ or CD11b^+^F4/80^+^TNF^+^ myeloid cells in the VAT of NeoATB mice (Supporting Information S1: Figure S6A,B). There was no significant change in the frequency and total number of IL-10^+^ CD11b^+^F4/80^+^ cells (Supporting Information S1: Figure S6A, B). The total number of B cells was not changed in NeoATB mice. However, the frequencies and numbers of CD19^+^IL-1β^+^, CD19^+^IL-10^+^, or double-positive CD19^+^IL-10^+^TNF^+^ B cells were increased in VAT of NeoATB mice compared to control mice (Supporting Information S1: Figure S6A,B). Consistent with inflammation in the VAT, 5-week-old NeoATB mice already had increased glucose intolerance (Figure 3F), although no insulin resistance was observed at this time point (Figure 3G). These data indicate that early exposure to antibiotics is associated with an increased proinflammatory immune response and reduction of Treg cells in the VAT of NeoATB mice detectable at 5 weeks of age.

### 2.4 | Defective Migration and Local Generation of Tregs in VAT of NeoATB Mice

**FIGURE 4 |:**
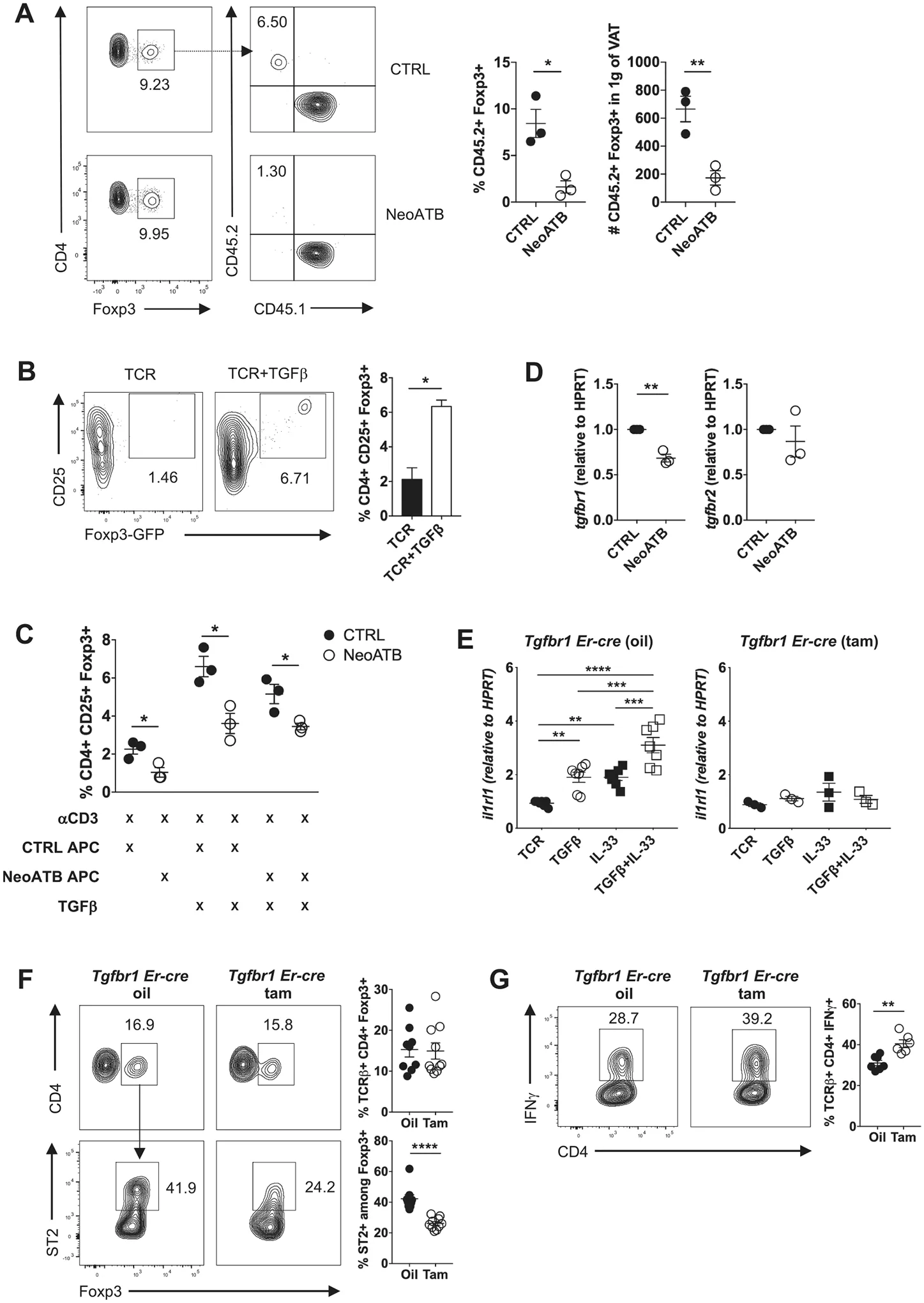
Defective migration and local generation of Tregs in VAT of NeoATB mice. (A) Tregs (Foxp3^+^) cells were sorted from spleens of Control (CD45.2^+^) or NeoATB (CD45.2^+^) mice and intraperitoneally injected into CD45.1^+^ mice. Treg migration was detected after 48 h by flow cytometry. The summarizing dot plot shows the frequency of adipose CD45.2^+^ Foxp3^+^ CTRL and NeoATB cells in the VAT of CD45.1^+^ mice. (B) Naïve T cells (CD4^+^CD25^−^GFP^−^) and antigen-presenting cells CD45^+^CD19^−^CD3^−^ (APCs) were sorted from the VAT of adult Foxp3^gfp^ male mice. Naïve T cells were co-cultured with APCs in the presence of 0.5 μg of anti-CD3 only or with/without 1 ng/mL of TGFβ for 72 h. The summarizing dot plot shows the frequency of CD4^+^CD25^+^Foxp3-gfp^+^ cells when cultured without TGFβ (TCR) or with TGFβ (TCR + TGFβ). (C) Naïve T cells (CD4^+^CD25^−^) and antigen-presenting cells CD45^+^CD19^−^CD3^−^ (APCs) were sorted from VAT of CTRL and NeoATB mice. CTRL naïve T cells were co-cultured either with CTRL or NeoATB APCs in the presence of 0.5 μg of anti-CD3 only or with/without 1 ng/mL of TGFβ for 72 h. NeoATB naïve T cells were co-cultured either with NeoATB or CTRL APCs in the presence of 0.5 μg of anti-CD3 only or with/without 1 ng/mL of TGFβ for 72 h. (D) Gene expression of *Tgfbr1* and *Tgfbr2* in the VAT of 5-week-old CTRL and NeoATB mice. (E) Treg cells were sorted from the spleen of *Tgfbr1 Er-cre* oil or tamoxifen-treated mice. Cells were stimulated with plate-coated 1 μg/mL of anti-CD3 and soluble 1 μg/mL of anti-CD28 in the presence of 10 ng/mL of IL2 and stimulated with 2 ng/mL of TGFβ or 10 ng/mL of IL-33 or a combination of both TGFβ + IL-33. Cells were cultured for 72 h, and *Il1rl1* mRNA was quantified by qPCR. (F) Representative FACS plots and dot plots show the frequency of CD4^+^Foxp3^+^ or ST2^+^ among Foxp3^+^ cells in the VAT of *Tgfbr1 Er-cre* oil or tamoxifen-treated mice. (G) Representative FACS plots and dot plots show the frequency of CD4^+^IFNγ^+^ cells in the VAT of *Tgfbr1 Er-cre* oil or tamoxifen-treated mice. Data are pooled from 2 to 3 or representative of one out of 2 (f, g) independent experiments. Significance was determined using a two-tailed Student *t*-test (*p < 0.05, **p < 0.01).

As Treg cells in the adipose tissue are critical in controlling the inflammation that dysregulates metabolism in adipocytes [9], we next investigated the mechanism by which Treg cells were reduced in the fat of NeoATB mice. As the current notion predicts that adipose Tregs migrate from the peripheral pool of thymically derived tTregs [19], we first addressed whether early Treg migration to adipose tissue could be impaired. Because purification of enough Treg cells from adipose tissue was technically challenging, we isolated splenic Tregs from both NeoATB and control mice before intraperitoneal injection into syngeneic CD45.1^+^ mice and assessment of homing 48 h later (see scheme of experiment in Supporting Information S1: Figure S7A). We found that Tregs from NeoATB mice exhibited significantly reduced migration to adipose tissue in the recipients compared to Tregs from control mice (Figure 4A), although both transferred Tregs showed no significant difference in the frequency in the spleens or lymph nodes of the recipient mice (Supporting Information S1: Figure S7B,C). Next, we studied whether neonatal antibiotic treatment could impact Treg conversion from naïve CD4^+^ T cells locally in the adipose tissues. As there remains a debate about whether Treg cells can be differentiated locally in the VAT [19], we first determined that Tregs could be converted from local CD4^+^CD25^−^Foxp3^−^ T cells. For this, we co-cultured VAT-sorted naïve CD4^+^CD25^−^ GFP^−^ (Foxp3^−^) T cells with VAT-derived APCs from Foxp3-GFP transgenic mice in the presence of soluble anti-CD3 and TGFβ for 3 days. We found that Foxp3-GFP^+^ cells were induced from CD4^+^ CD25^−^ GFP^−^ T cells stimulated with anti-CD3 and TGF-β (Figure 4B). Next, we compared local Treg conversion of VAT naïve CD4^+^ T cells between NeoATB and control mice. We observed a significantly reduced generation of Treg cells from VAT naïve T cells from NeoATB mice compared to control mice, irrespective of the presence of VAT-APCs (Antigen Presenting Cells) from NeoATB or control mice (Figure 4C). Of note, we also observed a slight but reproducible reduction in conversion to Treg cells when control naïve T cells were co-cultured with APCs sorted from VAT in NeoATB mice compared to control mice (Figure 4C), suggesting a functional change in NeoATB APCs as well. Mechanistically, naïve CD4^+^CD25^−^ T cells in the adipose tissue of NeoATB mice exhibited reduced *Tgfbr1* (encoding TGFβ receptor I) expression (Figure 4D), suggesting that defective TGFβ signaling in these VAT CD4^+^ T cells could underlie decreased Treg cell conversion [20]. These support the notion that a decrease in Treg cells in the VAT of NeoATB mice could be caused by a reduction of peripheral Treg recruitment or local proliferation as well as a defect in local Treg cell conversion within the VAT.

### 2.5 | TGF-β Maintains ST2 Expression in VAT Tregs

In addition to the defective migration and local conversion of Tregs in the VAT, we next investigated whether neonatal antibiotic treatment affected local Treg cell proliferation in the VAT. We focused on CD4^+^Foxp3^+^ST2^+^ Treg cells in the adipose tissue, as VAT Treg cells express ST2 (IL-33 receptor), which is vital for their proliferation in response to IL-33 [21]. NeoATB mice exhibited a significant decrease in ST2 expression in VAT Tregs (Figure 3D). We first determined that the levels of IL-33 protein in the VAT of five-week-old NeoATB mice were substantially reduced compared to control mice (Supporting Information S1: Figure S8A,B). We further identified that the decrease in IL-33 was mainly ascribed to CD45^−^ nonimmune cells in the VAT of NeoATB mice (Supporting Information S1: Figure S8C). In addition, a recent report demonstrated that the sex hormone androgen can regulate IL-33 production from the stromal cell population specific for male VAT and enhance the local expansion of Tregs [22]. We tested gene expression of androgen receptor (*Ar*) in the VAT of 5-week-old and 32-week-old male control and NeoATB mice. We detected significantly reduced expression of *Ar* in the VAT of 32-week-old NeoATB mice, although the decrease in *Ar* expression did not reach significance in the VAT of 5-week-old mice (Supporting Information S1: Figure S8D). To understand how ST2 was downregulated in adipose Tregs, we hypothesized that TGFβ signaling might play a role, as we have recently shown that TGFβ signaling controls ST2 expression in ILC2 cells [23]. To test this, we cultured purified *Tgfbr1*^−*/*−^ and control splenic Treg cells (*Tgfbr1*^*f/f*^ *ER-cre* mice treated with tamoxifen or oil, respectively) with TGFβ, IL-33, or TGFβ plus IL-33 in the presence of anti-CD3 and anti-CD28 and IL-2. We found that either TGFβ or IL-33 increased expression of *Il1rl1* (encoding ST2) compared to TCR stimulation alone in control (wild-type) Tregs (Figure 4E), and a combination of the two showed an additive effect (Figure 4E). However, deletion of the TGFβ receptor I completely abolished TGF-β-mediated *Il1rl1* increase (Figure 4E). Surprisingly, we found that the increase of *Il1rl1* expression driven by IL-33 or IL-33 plus TGFβ was also completely abrogated in *Tgfbr1*^−*/*−^ Tregs, suggesting that ST2 upregulation via IL-33 was TGFβ-dependent (Figure 4E). To directly test the effect of TGFβ on ST2 expression in VAT Tregs in vivo, we treated *Tgfbr1*^*f/f*^ *ER-cre* mice with tamoxifen or oil (Controls) for 5 days and examined the expression of ST2 directly in VAT Foxp3^+^ cells by flow cytometry. We showed that tamoxifen-treated *Tgfbr1*^*f/f*^ *Er-cre* mice exhibited a significantly reduced frequency of CD4^+^ ST2^+^ Foxp3^+^ Tregs in the VAT despite a comparable frequency of total Treg cells in the *Tgfbr1*^−*/*−^ VAT, confirming a role for TGFβ in maintenance of ST2 among Foxp3^+^ Treg cells in adipose tissue (Figure 4F). Similar to the phenotype observed in the VAT of 5-week-old NeoATB mice, *Tgfbr1*^*f/f*^ *ER-cre* mice treated with tamoxifen exhibited increased CD4^+^IFNγ^+^ Th1 cells in the VAT (Figure 4G). Collectively, these results indicate that TGFβ signaling maintains ST2 expression in VAT Treg cells to safeguard their expansion in response to IL-33.

### 2.6 | Increased Systemic LPS and Activation of VAT CD11c^+^MHCII^+^ Cells in NeoATB Mice

**FIGURE 5 |:**
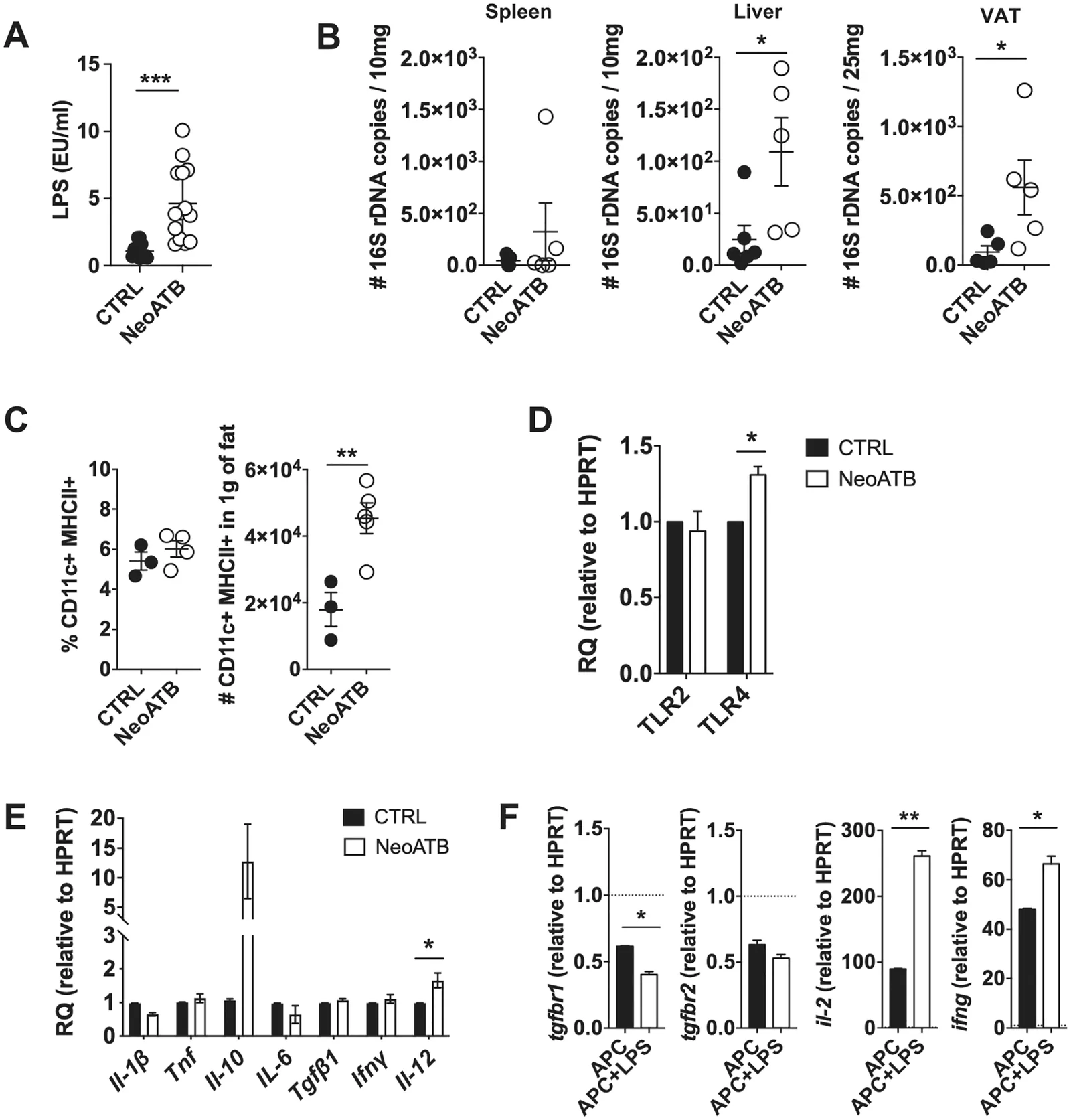
Increased systemic LPS and activation of VAT CD11c^+^MHCII^+^ cells. (A) Systemic levels of LPS tested in the serum of 5-week-old CTRL and NeoATB mice by ELISA. (B) Quantification of bacterial 16S rDNA copies in the spleen, liver, and VAT of 5-week-old CTRL and NeoATB mice determined by PCR. (C) Dot plots showing frequency and total number of CD11c^+^MHCII^+^ cells in the VAT of CTRL and NeoATB mice. (D) Gene expression of TLR2 and TLR4 in sorted CD11c^+^MHCII^+^ cells from the VAT of CTRL and NeoATB mice. (E) Gene expression of IL-1β, TNFα, IL-10, IL-6, TGFβ1, IFNγ, and IL-12 in sorted CD11c^+^MHCII^+^ cells from the VAT of CTRL and NeoATB mice. (F) Naïve T cells (CD4^+^CD25^−^) and antigen-presenting cells CD45^+^CD19^−^CD3^−^ (APCs) were sorted from VAT. APCs were stimulated for 6h with LPS (10 ng/mL) or left untreated. After 6 h, APCs were washed and co-cultured with naïve T cells in the presence of 0.5 μg/mL of anti-CD3. Cells were cultured o/n (~14h). The next day, CD4^+^ cells were sorted out from the culture, and *Tgfbr1*, *Tgfbr2*, *Il-2,* and *Ifng* mRNAs were quantified by qPCR. *Tgfbr1*, *Tgfbr2*, *Il-2,* and *Ifng* mRNAs in stimulated cells were compared to naïve T cells (dotted line = 1). Data are pooled from 2 (b–e) or 3 (a) or one out of 2 independent experiments (f). Significance was determined using a two-tailed Student *t*-test (*p < 0.05, **p < 0.01, ***p < 0.001).

One of the major drivers of obesity is linked to elevated plasma levels of lipopolysaccharide (LPS) or bacterial translocation in mice treated with a high-fat diet (HFD) [24–26]. It was also demonstrated that continuous subcutaneous low-rate infusion of LPS induced most metabolic disease abnormalities [25]. We hypothesized that the early onset of obesity observed in NeoATB mice could be linked to increased levels of LPS or low-grade bacteremia. We first determined elevated serum levels of LPS as well as the increased presence of 16S bacterial DNA in VAT and liver but not in the spleen of 5-week-old NeoATB mice (Figure 5A,B). In addition, we detected an increased total number of CD11c^+^MHCII^+^ cells in the VAT of NeoATB mice compared to control mice (Figure 5C; Supporting Information S1: Figure S9C), which could be mediated by the increased number of endogenous ligands (e.g., bacterial products). As toll-like receptors (TLRs), such as TLR2 and TLR4, induce insulin resistance, which is pivotal in the pathogenesis of obesity [27, 28], we found increased gene expression of TLR4 but not TLR2 in VAT CD11c^+^MHCII^+^ cells in NeoATB mice (Figure 5D). Moreover, we also observed significantly increased gene expression of *Il-12* among a panel of proinflammatory cytokines examined in CD11c^+^MHCII^+^ cells in VAT of NeoATB mice (Figure 5E). This was consistent with previous reports that DCs stimulated with LPS produced elevated levels of IL-12 [29]. Increased IL-12 can explain enhanced polarization of Th1 cells and IFNγ production in the VAT of NeoATB mice. We have previously shown that the strength of T cell receptor (TCR) stimuli determines *Tgfbr1* expression [20]. To test if CD11c^+^MHCII^+^ cells exposed to LPS may modulate *Tgfbr1* in naïve T cells, we sorted VAT APC (CD45^+^CD19^−^CD3^−^ cells) and stimulated them with or without LPS for 6 h. After extensive washes, VAT APCs were then co-cultured with VAT-sorted CD4^+^CD25^−^ cells for 14 h. CD4^+^ T cells were then FACS-sorted from the cultures and tested for the gene expression of *Tgfbr1*, *Tgfbr2*, *Il2*, and *Ifng*. We found that VAT CD4^+^ T cells co-cultured with LPS pre-stimulated VAT APCs exhibited a significant reduction in *Tgfbr1* but not *Tgfbr2*, and increased gene expression of *Il2* and *Ifng* (Figure 5F). Thus, increased activation of VAT CD11c^+^MHCII^+^ cells via the LPS-TLR4 pathway downregulates TGFβ receptor I in naïve CD4+ T cells, providing an underlying mechanism for the decrease in *Tgfbr1* in CD4^+^CD25^−^ T cells that results in the defect of local Treg conversion in VAT of NeoATB mice (Figure 4C,D).

### 2.7 | Increased Intestinal Permeability in Young NeoATB Mice

**FIGURE 6 |:**
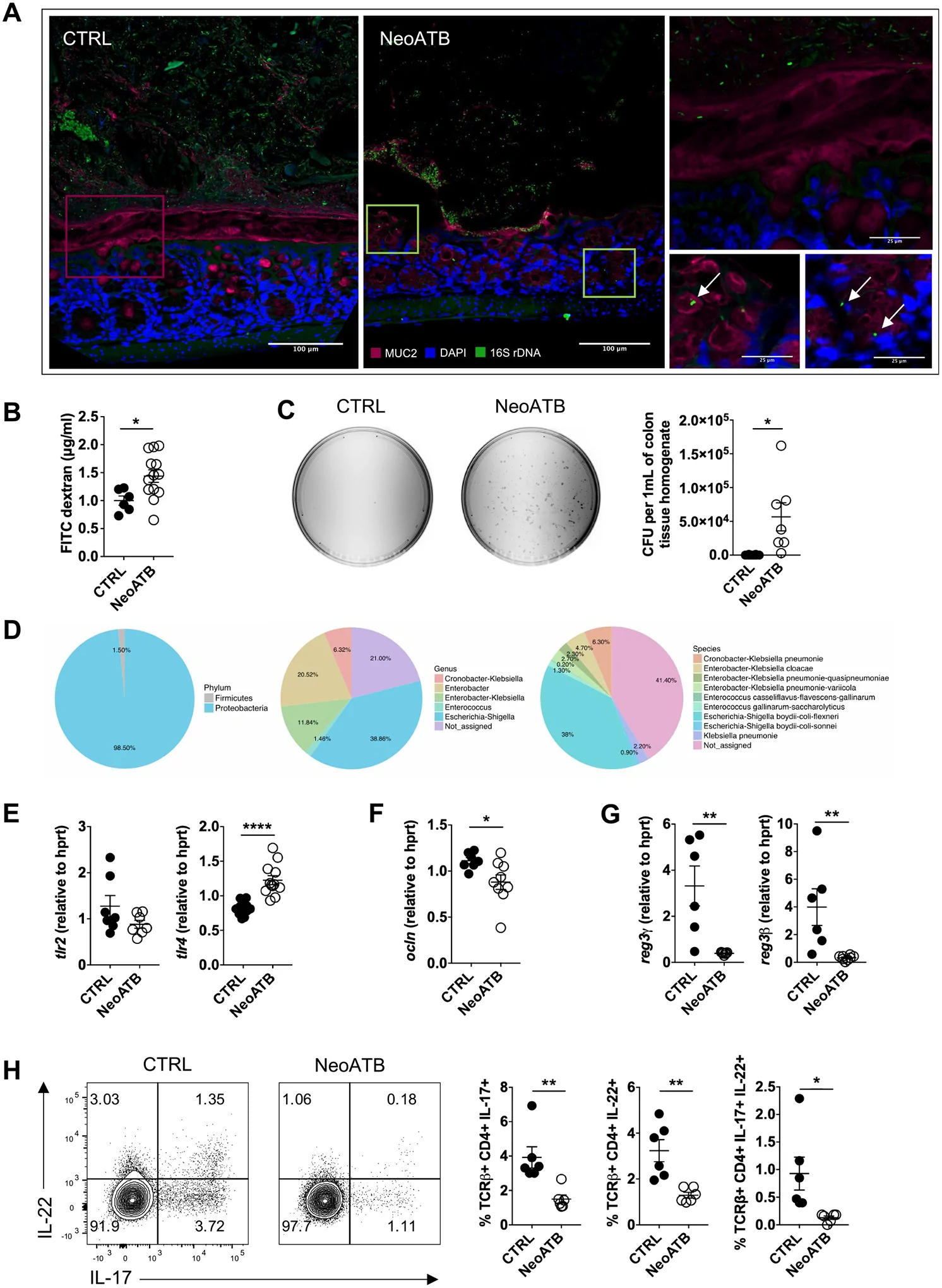
Increased intestinal permeability in the colon of 5-week-old NeoATB mice. (A) Fluorescence in Situ Hybridization of 16S rRNA tested by confocal microscopy in the colon of 5-week-old CTRL and NeoATB mice. The upper right panel represents the microbiota/mucus layer region in CTRL mice. The two lower right panels show zoomed-in areas showing bacterial translocation in the colon of NeoATB mice. (B) Intestinal permeability between CTRL and NeoATB mice was tested using a FITC-dextran assay. (C) Colon homogenates from CTRL and NeoATB mice plated on Luria-Bertani agar plates are shown as CFU per 1 mL of colon homogenate. (D) 16S sequencing analysis of microbiota from the colon homogenate of NeoATB mice. (E) Gene expression of TLR2 and TLR4 in the colon of 5-week-old CTRL and NeoATB mice. (F) Gene expression of occludin (*ocln*) in the colon of 5-week-old CTRL and NeoATB mice. (G) Gene expression of *reg3γ* and *reg3β* in the colon of 5-week-old CTRL and NeoATB mice. (H) Representative FACS plots showing frequency of TCRβ^+^CD4^+^IL-17^+^, TCRβ^+^CD4^+^IL-22^+,^ and double-positive TCRβ^+^CD4^+^IL-17^+^IL-22^+^ cells in the colon of 5-week-old CTRL and NeoATB mice. Data is pooled from 2 to 3 independent experiments. Significance was determined using a two-tailed Student *t*-test (*p < 0.05, **p < 0.01, ****p < 0.0001).

To elucidate the mechanisms responsible for increased systemic LPS and VAT bacterial detection and inflammation in NeoATB mice, we hypothesized increased permeability in the colon of the antibiotics-treated mice, as the colon harbors the highest number and diversity of microbiota. To study intestinal permeability, we utilized FISH hybridization (panbacterial 16S probe; green) to visualize bacteria in the lumen of the colon in control and NeoATB mice at an early age (5-week-old). In contrast to control mice, NeoATB mice exhibited reduced luminal bacterial content in the colon (green) and decreased thickness of the mucus layer (red), but surprisingly increased bacterial translocation into the epithelial layer (blue) (Figure 6A). To confirm the increased intestinal permeability in NeoATB mice, we utilized the FITC-dextran assay. Consistent with the FMT experiment, there was a significant increase in plasma levels of FITC-dextran in NeoATB compared to control mice (Figure 6B). To further investigate increased intestinal permeability, the colon lumens of control and NeoATB mice were extensively washed to remove all luminal content, and colon tissue homogenates were plated overnight on LB agar plates to examine bacterial growth. Consistent with FISH hybridization results, we detected growing bacterial colonies only in colon homogenates from NeoATB mice but not control mice (Figure 6C), suggesting translocation of the bacteria in the colon tissue. 16S sequencing analysis revealed that the majority (98.5%) of translocated bacteria belong to the phylum *Proteobacteria* (e.g., *Klebsiella, Shigella, Enterococcus,* and *Escherichia* species) (Figure 6D). Consistent with 16S sequencing results, in the colon of NeoATB mice, we observed significantly increased expression of TLR4 but not TLR2 (Figure 6E). It was suggested that increased intestinal permeability may involve reduced expression of epithelial tight junction proteins [25]. In the colon of NeoATB mice, we detected decreased gene expression of the tight junction protein occludin, but not zonulin or claudin (Figure 6F, data not shown). Furthermore, we found significantly reduced gene expression of both *Reg3γ* and *Reg3β* in the colon of NeoATB mice (Figure 6G), encoding two antimicrobial proteins (AMPs) that are crucial for intestinal barrier defense [26]. As intestinal barrier integrity is also dependent on the intestinal immune cells such as IL-22-producing T cells [30], we found a significant reduction in intestinal CD4^+^IL-22^+^ (Th22) and CD4^+^IL22^+^IL-17^+^ cells in the colon lamina propria of 5-week-old NeoATB mice (Figure 6H). Intestinal CD4^+^IL-17^+^ IL-22^−^ (Th17) cells were also significantly reduced in the NeoATB mice (Figure 6H). The data collectively indicate increased intestinal permeability in the colon of NeoATB mice, which was mediated by dysregulation of epithelial and immunological factors, including Th22 cells.

## 3 | Discussion

The incidence of many common multifactorial human diseases, such as obesity, diabetes, allergy/asthma, and inflammatory bowel disease, has substantially increased during the past two centuries. The short duration of this period, which encompasses only a limited number of human generations, makes it unlikely that these diseases can be explained by genetic factors alone. Instead, changes in lifestyle and environmental factors are probably associated with the increasing incidence of inflammatory and metabolic diseases [31]. In recent years, many multifactorial diseases have shown that an increasing incidence of inflammatory and metabolic diseases has been associated with altered microbiota compositions, termed dysbiosis. The overall goal of our series of experiments was to elucidate potential immunological mechanisms by which neonatal dysbiosis increases the risk of development of obesity and associated metabolic disorders later in life.

### 3.1 | NeoATB Treatment Drives Obesity With Decreased Tregs and Increased Th1 Inflammation in the VAT

Microbiota dysbiosis was induced by antibiotics vancomycin (targets gram-positive bacteria) and polymyxin B (targets gram-negative bacteria). Both antibiotics are poorly absorbed in the gut, thus reducing systemic effects. For more than 5 decades, we have known that the administration of antibacterial agents promotes the growth of farm animals [32]. These effects are broad across vertebrate species and routes of administration, either in feed or water, and predispose microbiota as a primary target. Early life microbiota undergoes dynamic changes, with progressive stabilization to an adult-like community structure after 3 years of age [33]. It was shown that subtherapeutic antibiotic treatment at a young age (started at 4 weeks) had a significant effect on the development of fat mass but not body weight in mice [16]. Recently, it was demonstrated that low-dose penicillin (LDP) treatment during the neonatal age (interval 0–4 weeks) can significantly enhance HFD-induced obesity [34]. In our experiments, we observed that antibiotic treatment (vancomycin + polymyxin B) during the first 3 weeks of life (neonatal age, NeoATB), without any further treatment, was sufficient to boost the development of obesity in adulthood. Unexpectedly, as early as 5 weeks of age, we already observed the onset of obesity, which worsened in adult mice. Five-week-old NeoATB mice already have increased body weight and adipose weight and increased glucose intolerance, albeit no difference in insulin resistance. They started to display the first signs of adipose tissue inflammation, represented by decreased Treg cells and increased Th1-IFN-producing cells.

### 3.2 | NeoATB Treatment Leads to Permanent Dysbiosis of Gut Microbiota

Administration of antibiotics during the first 3 weeks of life caused microbiota alteration when the mice were 5 weeks old. More interestingly, microbiota dysbiosis was long-lasting and still present in adult mice. The development of obesity has been linked to changes in the *Firmicutes* to *Bacteroides* ratio. The relative proportion of *Bacteroidetes* was reported to decrease in obese people compared with lean people [35]. Increased abundance of *Firmicutes* in obese people was linked with an increased capacity to harvest energy from the diet, thus promoting more efficient absorption of calories and subsequent weight gain [36]. In experimental models of obesity, a greater ratio of *Firmicutes* to *Bacteroidetes* has been confirmed in genetically or diet-induced obese mice relative to controls [37]. Consistent with this notion, NeoATB mice indeed exhibit significantly increased phylum *Firmicutes* and significantly reduced abundance of phylum *Bacteroidetes,* resulting in a higher ratio of *Firmicutes* to *Bacteroidetes* compared to control mice. In addition, the microbiota composition in NeoATB mice was significantly different from the microbiota of control mice (beta diversity). Phylum *Verrucomicrobia* has been linked with a positive effect on body weight and metabolic disease [38]. In contrast, in our studies, neonatal antibiotic treatment also increased the abundance of the phylum *Verrucomicrobia,* and this trend became stable through adulthood. Using FMT experiments, we have confirmed that the altered microbiota of NeoATB mice are responsible for increased intestinal permeability and the development of obesity. Antibiotic treatment, per se, was insufficient to increase intestinal permeability or potentiate the obesity phenotype.

### 3.3 | NeoATB Treatment Results in Increased Intestinal Permeability

Obesity has been linked with increased intestinal permeability [26], and we discovered that transient neonatal antibiotic use results in increased intestinal permeability, which was already evident at 5 weeks of age. We identified several mechanisms responsible for increased intestinal permeability in NeoATB mice. Firstly, in 5-week-old NeoATB mice, we detected a significant reduction of SCFAs, likely due to a reduction in bacterial load and taxonomic diversity in the gut of NeoATB mice. As butyrate is an important energy source for intestinal epithelial cells, decreased levels of butyrate may reflect a thinner epithelial layer in the colon of NeoATB mice [39]. Secondly, neonatal antibiotic treatment suppressed the expression of antimicrobial proteins such as Reg3γ and Reg3β as well as reduced expression of tight junction protein occludin in the gut. Lastly, the significant decrease in IL-17 and IL-22-producing T cells, especially Th22 cells in NeoATB mice may further reduce barrier integrity and defense against pathogens [30]. As a result of increased permeability of the intestinal barrier in NeoATB mice, we observed increased translocation of bacteria in the colon, where the majority (98.5%) belonged to the phylum *Proteobacteria*. *Proteobacteria* represent gram-negative bacteria containing LPS in the outer membrane. A connection between low-grade inflammation, sustained by LPS, and the development of metabolic disorders is well established [40]. Consistently, we found increased serum levels of LPS and enhanced influx of bacterial DNA in the VAT and liver of NeoATB mice. A similar observation was made in mice treated with HFD [24]. The increased circulating levels of LPS and translocation of bacteria into distant tissues such as the liver and VAT will inevitably result in a higher degree of inflammation in target tissues. Indeed, we found an increased number and activation of CD11c^+^MHCII^+^ cells, as evidenced by increased TLR4 expression (but not TLR2) and significantly increased IL-12 gene expression in the VAT of NeoATB mice. IL-12-producing CD11c^+^MHCII^+^ cells might increase Th1 cell differentiation in addition to the decreased Tregs, contributing to the increased VAT inflammation and dysregulation of adipocyte metabolism.

### 3.4 | NeoATB Treatment Decreases the Infiltration, Local Proliferation, and Generation of Tregs in VAT

Treg cells in the VAT constitute a population of tissue-resident cells that are crucial for preserving metabolic homeostasis and limiting the development of obesity-induced insulin resistance [9]. We have revealed here that the decrease in Tregs in the VAT of NeoATB mice is due to the combinational effects of reduced peripheral infiltration and local proliferation and conversion in the adipose tissues. Recent reports have shown that VAT Treg cells express markers such as neuropilin1 (NRP1) and Helios, and therefore they may originate from the thymus, and their accumulation depends on in situ recognition of an MHCII-restricted antigens and requires the presence of IL-33 for their expansion [19]. Until now, no evidence exists about the local conversion of VAT naïve T cells to Treg cells. We discovered that VAT-naïve CD4^+^CD25^−^ T cells can be converted to CD4^+^CD25^+^Foxp3^+^ Treg cells in the presence of TGFβ. VAT CD4^+^CD25^−^ Foxp3^−^ T cells isolated from NeoATB mice exhibited reduced capacity for conversion to Treg cells in vitro, which underlies the decrease in VAT Tregs in these obese mice. We elucidated that the reduction of TGFβ signaling in the VAT CD4^+^CD25^−^ T cells of NeoATB mice was attributed to the downregulation of *tgfbr1* in these T cells. As we have previously shown that the strength of TCR stimuli determines *Tgfbr1* expression in T cells [20], we further showed that VAT naïve T cells cocultured with LPS-pretreated VAT CD11c^+^MHCII^+^ cells (mimicking the environment in the VAT of NeoATB mice) significantly downregulated their expression of *Tgfbr1*. This explains the downregulation of *tgfbr1* expression in freshly isolated CD4^+^CD25^−^ T cells in VAT of NeoATB mice.

The reduction of VAT Treg cells in NeoATB mice could also be explained by the defective Treg expansion due to the malfunction in their IL-33-ST2 signaling. Normal VAT Treg cells express high levels of the IL-33 receptor ST2, which is essential for IL-33-driven Treg cell expansion. Both ST2^−/−^ and IL-33^−/−^ mice showed reduced frequency of Treg cells in VAT but not in the spleen or lymph nodes [21]. Our findings suggest that there is a decrease in the levels of IL-33 protein and expression of IL-33 in CD45^−^ adipose stromal cells. It is important to note that stromal adipose cells were considered a primary source of IL-33. ST2 may also be regulated by PPAR,γ since VAT Treg cells lacking PPARγ show a lower percentage of ST2^+^ cells [41]. We found the decreased frequency of ST2 among Foxp3^+^ cells in the VAT in 5-week-old NeoATB mice. Unexpectedly, we discovered the importance of TGFβ signaling in the maintenance of ST2 in VAT Tregs. Support for this conclusion includes that not only TGFβ treatment upregulate ST2 expression in Tregs, but surprisingly, the IL-33-mediated increase of ST2 in Treg cells is also TGFβ dependent. Furthermore, depletion of *Tgfbr1* in tamoxifen-treated *Tgfbr1*^*f/f*^ *Er-cre* mice revealed dramatically reduced expression of ST2 in their VAT Treg cells. Importantly, the frequency of VAT Tregs was unchanged between tamoxifen- and oil-treated *Tgfbr1*^*f/f*^ *ER-cre* mice, most likely due to a short period of time (5 days) needed for *Tgfbr1* depletion. Consistently, these *Tgfbr1* knockout mice, like NeoATB mice, had increased frequencies of CD4^+^IFNγ^+^ cells in adipose tissue. The measurement of active TGFβ1 in the tissues is difficult and impractical because TGFβ activation can be affected by many factors (e.g., physical, chemical, or enzymatic), and the tissue process could nonspecifically affect the activation of TGFβ, which might result in incorrect interpretation of the data; we instead have used mice with *Tgfbr1* deletion to study the TGFβ signal function. Notably, adult obese NeoATB male mice have reduced gene expression of androgen receptor in the VAT, which in part can explain the reduced frequency of Tregs in the VAT of these mice [22]. We only used male mice since the obesity phenotype was more stable in males than in females. Therefore, we did not compare androgen receptor expression between male and female mice. As a result of the reduction of Tregs in VAT, Th1-type inflammation was increased, and consequently, the NeoATB mice developed a significant obesity phenotype with metabolic abnormalities such as insulin resistance or glucose intolerance. Consistent with our data in mice, it was reported that infants exposed to antibiotics during the first 6 months of life had increased body mass by 10–38 months, whereas exposures later in infancy (6–14 or 15–23 months) were not consistently associated with increased body mass [42].

## 4 | Conclusion

Collectively, our findings provide evidence that disruption of gut microbial communities during the first 3 weeks of life represents a key factor leading to the development of obesity in adulthood. Dysbiosis of microbiota caused increased intestinal permeability and diminished TGFβ signaling in the VAT, which resulted in a decrease in Tregs and an increase in Th1-type inflammation, leading to an alteration of the adipose tissue immune responses and consequently obesity and metabolic abnormalities. Our data also indicate that the obesity phenotype correlates with an absence of phylum *Bacteroidetes* and an increase in the abundance of phylum *Firmicutes*. Consistent with our data, the increased ratio between *Firmicutes* and *Bacteroidetes* was found in gut microbiota in the human population with obesity [35]. Thus, extreme attention must be paid to prevent unnecessary antibiotic use in the neonatal age to avoid permanent alteration of the gut microbiota and, consequently, metabolic abnormalities and obesity in adulthood.

### 4.1 | Limitations of the Study

We are aware of 2 limitations: first, all experiments shown in the manuscript were done in male mice, and all experiments utilizing adipose tissue were done in epididymal adipose fat depots. On the other hand, another study confirmed the development of obesity in female mice after neonatal treatment with a low dose of penicillin. However, this study did not investigate the function/phenotype of fat Treg cells in female mice [34].

Second, the manuscript did not investigate other factors contributing to obesity, such as a high-fat diet or a combination of neonatal-age antibiotics plus a high-fat diet.

### 4.2 | Experimental Procedures

#### 4.2.1 | Mice

C57BL/6J, Foxp3^gfp,^ and Tgfbr1^f/f^ Er-Cre male mice were used in experiments. Mice were bred under specific pathogen-free conditions in the animal facility of the National Institute of Dental and Craniofacial Research. All animal studies were performed according to National Institutes of Health guidelines for the use and care of live animals and were approved by the Animal Care & Use Committee (ACUC) of the National Institute of Dental and Craniofacial Research. We have complied with all relevant ethical regulations for animal testing and research.

#### 4.2.2 | Mice Treatments

Neonatal antibiotic treatment was performed using Vancomycin hydrochloride (500 mg/L, Hospira) and Polymyxin B (1000000U/l X-gen). Neonates were treated in intervals of 3 weeks and started immediately after birth. After 3 weeks, mice were weaned and housed in a normal environment with normal food and water. The weights of the mice were monitored during the experiment. Tissues were harvested at the end of the experiments for histopathological and immunological analyses.

### 4.3 | ELISA

The levels of IL-33 in the tissue supernatants were assayed using sandwich Enzyme-Linked Immunosorbent Assay (ELISA) kits as per the manufacturer’s instructions (Invitrogen by Thermo Fisher Scientific). Briefly, a standard 96-well plate (Nunc-Immuno MaxiSorp Uncoated Plates) was coated with appropriate capture antibody and incubated at 4°C overnight. After washing, the nonspecific binding was blocked by adding assay diluent. Standards and tissue supernatants were dispensed into the wells, and after 1.5 h of incubation, the detection antibody was added into each well for 1 h. After washing, avidin-HRP enzyme conjugate was added into each well. 30 min later, the plate was washed and incubated with TMB substrate solution at room temperature in the dark until an adequate signal developed. The reaction was stopped by 2N H_2_SO_4_, and the optical density was determined using the microplate reader Spectramax Plus (Molecular Devices, San Jose, CA). The concentration of IL-33 in each sample was interpolated from standard curves and expressed as picograms of cytokine per mL. Serum levels of LPS were determined using ELISA (LSBio).

### 4.4 | Glucose Tolerance Test and Insulin Tolerance Test

Mice underwent metabolic studies beginning at about 7 days before the end of experiments, when the glucose tolerance test (GTT) was performed. Mice were fasted overnight (15–16 h), weighed, and then evaluated for blood glucose concentrations at 0, 15, 30, 60, 90, and 120 min after intraperitoneal glucose administration (2 g/kg). The insulin tolerance test (ITT) was performed ~4 days after the GTT. For ITT, mice were fasted for 4–6 h and injected intraperitoneally with insulin (Humulin R, Lilly; 0.75 U/kg). Glucose concentration was determined at 0, 20, 40, 60, and 80 min afterward.

### 4.5 | Fecal Microbiota Transplantation

Fecal samples were resuspended in ice-cold reducing phosphate-buffered saline (PBS) (containing 0.05% L-Cysteine hydrochloride, Sigma-Aldrich) (100 mg/mL). After homogenization and removal of large particles by centrifugation, the fecal bacteria suspension was filtered through a 70 μm cell strainer. All GF mice were pooled together and randomly assigned to different groups. The GF mice were orally gavaged with 200 μL of bacterial suspension once a week for 2 weeks.

### 4.6 | Intestinal Permeability Testing

Mice were fasted for 6 hours and then gavaged with 60 mg/kg FITC-Dextran. After 4 hours, sera were collected and centrifuged at 15,000 g for 15 min. Serum FITC-Dextran levels were determined with 490 excitation/520 emission by Cytation 5.

### 4.7 | Preparation and Flow Cytometry of Adipose Tissue Samples

Mice were euthanized using CO_2_. Epididymal adipose tissue was removed and placed into Dulbecco’s modified Eagle’s medium (DMEM), mechanically disaggregated by scissors, and incubated with collagenase type II (1 mg/mL; Worthington Biochemical Corporation) at 37°C for 45 min. Single-cell suspensions were obtained by mincing the tissues through 70-μm cell strainers and further washed with DMEM. For flow cytometry involving surface staining only, cell preparations were subjected to red blood cell lysis by incubating with 1 mL ammonium–chloride–potassium lysing (ACK) buffer for 2–3 min on ice, washed, and finally resuspended in DMEM. Live/dead staining was done prior to surface staining using Zombie Yellow Fixable Viability Kit (BioLegend) for 10 min at room temperature. Surface staining with anti-mouse-specific antibodies was done for 20 min at 4°C. Following standard surface staining, intracellular staining of transcription factors was done using FOXP3/Transcription Factor Staining Buffer Set (Thermo Fisher Scientific) followed by staining with anti-mouse-specific antibodies (such as anti-mouse Foxp3 or anti-mouse Gata3). For intracellular cytokine staining, cells were stimulated for 4 h at 37°C with PMA (phorbol 12-myristate 13-acetate; 5 ng/mL), Ionomycin (1 μg/mL), and Golgi-Plug (1:1000 dilution; BD Pharmingen), followed by BD Cytofix/Cytoperm permeabilization buffer according to the manufacturer’s instructions (BD Biosciences). Cells were further stained with specific antibodies to determine cytokine expression.

### 4.8 | PCR Quantification

RNA from adipose tissue was extracted using the protocol described previously [43]. RNA from ex vivo sorted adipose T cells or CD11b^+^F4/80^+^ cells was extracted using RNeasy Plus Micro Kit (Qiagen). cDNA was synthesized using a High-Capacity cDNA Reverse Transcription Kit (Applied Biosystems by Thermo Fisher Scientific). In samples with low concentrations of RNA/cDNA (mostly from sorted cells), preamplification was performed using TaqMan PreAmp Master Mix (Applied Biosystems by Thermo Fisher Scientific). qPCR was done using TaqMan assays following manufacturer instructions. All TaqMan assays are listed in the Supporting Information S1: Table S1. Total transcript values were normalized to those from the mouse *Hprt* gene. Results were calculated using the comparative ΔΔCT method.

### 4.9 | In Vitro T Cell Cultures

Naïve T cells (CD4^+^CD25^−^GFP^−^) and antigen-presenting cells CD45^+^CD19^−^CD3^−^ (APCs) were sorted from VAT of adult Foxp3^gfp^ male mice. Naïve T cells were co-cultured with APCs in the presence of 0.5 μg of anti-CD3 only or with/without 1 ng/mL of TGFβ for 72h. Alternatively, Naïve T cells (CD4^+^CD25^−^) and antigen-presenting cells CD45^+^CD19^−^CD3^−^ (APCs) were sorted from VAT of CTRL and NeoATB mice. CTRL naïve T cells were co-cultured either with CTRL or NeoATB APCs in the presence of 0.5 μg of anti-CD3 only or with/without 1 ng/mL of TGFβ for 72 h. NeoATB naïve T cells were co-cultured either with NeoATB or CTRL APCs in the presence of 0.5 μg of anti-CD3 only or with/without 1 ng/mL of TGFβ for 72 h. To test Treg cell expression of *il1rl1* (ST2), Treg cells were sorted from the spleen of *Tgfbr1 Er-cre* oil = or tamoxifen-treated mice. Cells were stimulated with plate-coated 1 μg/mL of anti-CD3 and soluble 1 μg/mL of anti-CD28 in the presence of 10 ng/mL of IL-2 and stimulated with 2 ng/mL of TGFβ or 10 ng/mL of IL-33 or a combination of both TGFβ + IL-33. Cells were cultured for 72 h, and expression of *Il1rl1* mRNA was quantified by qPCR. To test the strength of DC cell stimulation on naïve T cells, Naïve T cells (CD4^+^CD25^−^) and antigen-presenting cells CD45^+^CD19^−^CD3^−^ (APCs) were sorted from VAT. APCs were stimulated for 6 h with LPS (10 ng/mL) or left untreated. After 6 h, APCs were washed and co-cultured with naïve T cells in the presence of 0.5 μg/mL of anti-CD3. Cells were cultured o/n (~14 h). The next day, CD4^+^ cells were sorted out from the culture, and expression of *Tgfbr1*, *Tgfbr2*, *Il-2,* and *Ifng* mRNAs was determined using qPCR.

### 4.10 | Confocal Microscopy

For FISH, the colon tissue was prepared by fixation in 60% methanol, 30% chloroform, and 10% glacial acetic acid, washed in 70% ethanol, and embedded in paraffin. Longitudinal sections (5–25 μm) were hybridized to a bacterial 16S rRNA gene probe: [AminoC6 + Alexa488] GCTGCCTCCCGTAGGAGT [AmC7 Q + Alexa 488] (Eurofins MWG Operon) as previously published [44] and counterstained with MUC2 and DAPI.

### 4.11 | 16S Sequencing

The total bacterial load was determined via qPCR using 16S rRNA gene primers, and *Streptococcus gordonii* (ATCC 35105) genomic DNA was used as a standard. The V4 region of the 16S rRNA gene was amplified from fecal gDNA. According to the manufacturer’s instructions, DNA was extracted using the QIAamp DNA Stool Mini Kit (Qiagen). Sequencing was performed on the Illumina MiSeq instrument. The Nephele platform [45] (https://nephele.niaid.nih.gov) was used to demultiplex and cluster sequences into 97% identity OUT’s (QIIME pipeline) and assign taxonomic annotation based on the open Greengene database. Abundance, diversity plots, and LEfSe (LDA) were calculated using the Microbiome Analyst project [46, 47]. All plots showing microbiota abundance and diversity were generated in R [48].

### 4.12 | Chromatographic and Mass Detection Analysis

Short-chain fatty acid (SCFA) -acetate, -propionate, and -butyrate chromatography was carried out on a capillary column (30 m × 0.250 mm, 0.25 μm; Agilent Technologies) with 34.5 min run time. SCFA were separated at 2.61 min retention time, m/z 60 (qualifier ions m/z 43, 29) for acetate; 2.65 min retention time, m/z 74 (qualifier ions m/z 57, 45) for propionate; 2.71 min retention time, m/z 60 (qualifier ions m/z 88, 73) for butyrate; protonated forms were measured on SIM mode of 20–550 m/z. Analyses were performed with an Agilent 6890N gas chromatograph coupled to an Agilent 5973 mass-selective detector (MSD) under the following chromatographic conditions: Initial temperature 100°C for 0.50 min, increasing to 180°C at 8°C/min for 21 min and finally increased 200°C at 20°C/min for 2 min. The front inlet temperature was 240°C, operating with a split-less operation mode. MSD ion source and interface temperatures were 230°C, and the MS Quad at 150°C. The MSD operated in EI mode at 70 Ev and 1306 relative EMV volts. Carrier gas was He (1.0 mL/min). GC-MS data were acquired and processed using Agilent MassHunter WorkStation Software.

### 4.13 | Sample Preparation for GC-MS Analysis

Fecal pellets were placed into 0.3 mL of deionized water and followed by homogenization using a Precellys homogenizer (Bertin Instruments), using 1.0 mm zirconia/silica beads for 3 × 40 sec at 6500 rpm. The pH of the suspension was adjusted to 2.2 ± 0.05 by adding 5M HCl at room temperature for 20 min with sonication. The samples were centrifuged at 20000 g for 15 min at 4°C. Furthermore, 0.2 mL of supernatant was transferred into a GC-MS vial and 2 μL injected into the GC-MS using an autosampler. Samples were analyzed as above in GC-MS. Final concentrations were normalized to the weight of fecal pellets.

### 4.14 | Histology

Adipose or liver tissues were collected and fixed in 10% Neutral Buffered Formalin. All tissues were processed at the Histoserv company (https://www.histoservinc.com). Two sections per tissue were stained with hematoxylin and eosin for analysis. Images were acquired with the Leica Aperio Scan Scope instrument and analyzed using Aperio Image Scope Software. Adipocyte size was determined using Adiposoft Software [49].

### 4.15 | Statistical Analysis

All statistical analyses were performed using GraphPad Prism 8 software. All data are presented as mean ± S.E.M. Statistical significance, as indicated by asterisks, was determined by an unpaired *t*-test (two-tailed). *p* < 0.05 was considered significant (*, *p* < 0.05; **, *p* < 0.01; ***, *p* < 0.001; ****, *p* < 0.0001).

## Supplementary Material

Supplementary Materials

Additional supporting information can be found online in the Supporting Information section.

**Supporting Information S1:** dni270003-sup-0001-suppl-data.pptx.

## Data Availability

The data that support the findings of this study are available from the corresponding author upon reasonable request.
