# Supplementary material for: Neonatal Exposure to Antibiotics Triggers Obesity in Adulthood by Permanent Microbiota Dysbiosis and Reduction of Adipose Regulatory T Cells
Source: Barrier Immun. Author manuscript; Available in PMC 2026 Sep 29. (PMC13618908; doi:10.1002/dni2.70003)
Supplement: Supplementary file 1 [file NIHMS2211396-supplement-Supplementary_Materials.pdf]

SUPPLEMENTARY FIGURES AND TABLES

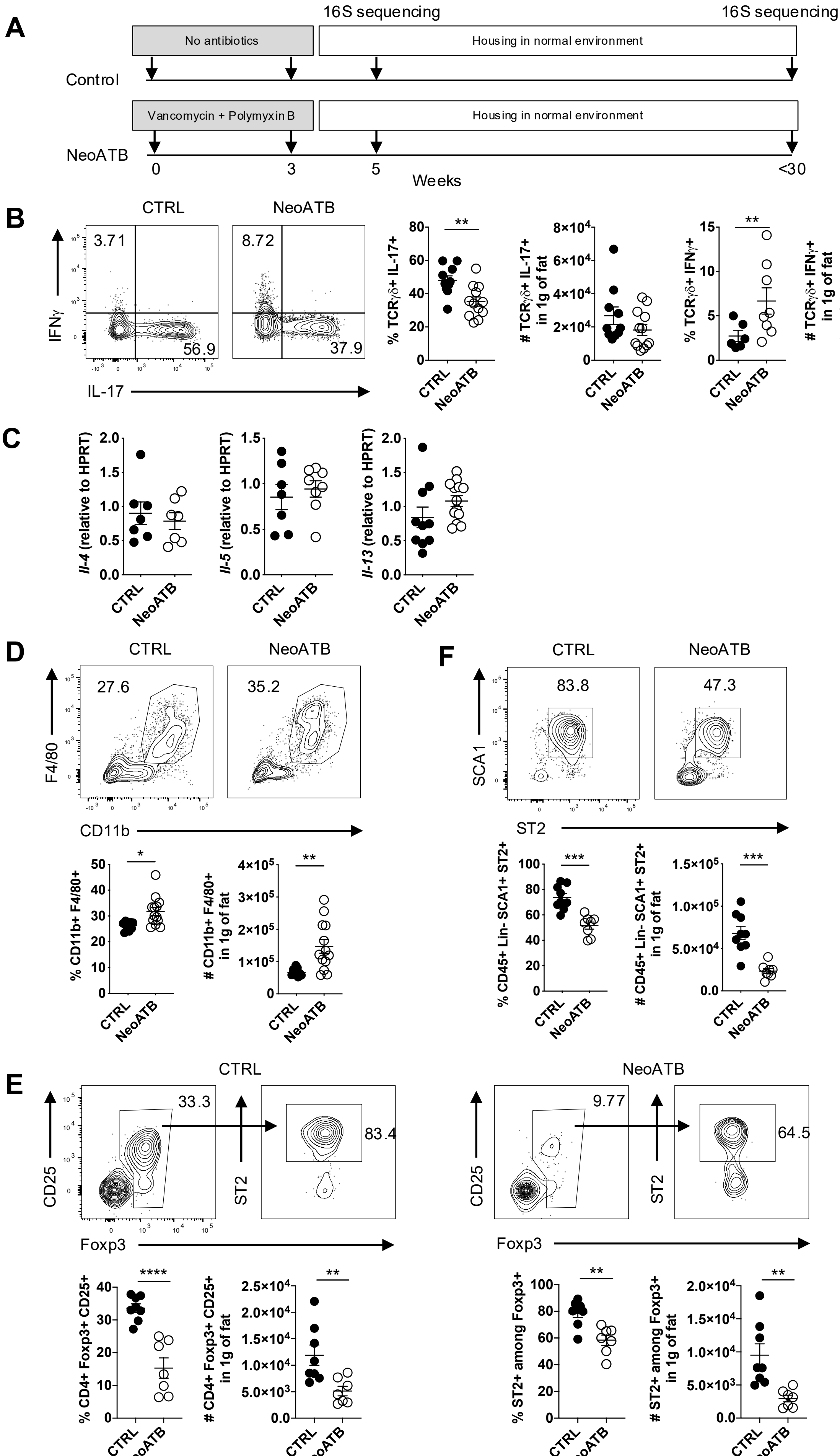
A
16S sequencing
16S sequencing
No antibiotics
Housing in normal environment
Control
Vancomycin + Polymyxin B
Housing in normal environment
NeoATB
0
3
5
<30
Weeks
B
CTRL
NeoATB
IFNγ
IL-17
3.71
56.9
8.72
37.9
% TCRγδ+ IL-17+
# TCRγδ+ IL-17+ in 1g of fat
% TCRγδ+ IFNγ+
# TCRγδ+ IFNγ+ in 1g of fat
C
Il-4 (relative to HPRT)
Il-5 (relative to HPRT)
Il-13 (relative to HPRT)
D
CTRL
NeoATB
F4/80
CD11b
27.6
35.2
% CD11b+ F4/80+
# CD11b+ F4/80+ in 1g of fat
F
CTRL
NeoATB
SCA1
ST2
83.8
47.3
% CD45+ Lin- SCA1+ ST2+
# CD45+ Lin- SCA1+ ST2+ in 1g of fat
E
CTRL
NeoATB
CD25
Foxp3
ST2
33.3
83.4
9.77
64.5
% CD4+ Foxp3+ CD25+
# CD4+ Foxp3+ CD25+ in 1g of fat
% ST2+ among Foxp3+
# ST2+ among Foxp3+ in 1g of fat

Figure S1

Altered immune profile in adipose tissue of adult NeoATB mice.

A. Immunization scheme
B. Representative FACS plots showing the frequency of $TCR\gamma\delta^+IL\text{-}17^+$ or $TCR\gamma\delta^+IFN\gamma^+$ cells in adipose tissue of CTRL and NeoATB mice. Dot plots showing summarized frequencies and total numbers of $TCR\gamma\delta^+IL\text{-}17^+$ or $TCR\gamma\delta^+IFN\gamma^+$ cells in adipose tissue of adult (~30 weeks old) CTRL and NeoATB mice.
C. Gene expression of Th2 cytokines *Il-4*, *Il-5,* and *Il-13* in the adipose tissue of CTRL and NeoATB mice.
D. Representative FACS plots showing the frequency of macrophages (gated as $CD11b^+F4/80^+$) in adipose tissue of CTRL and NeoATB mice. Dot plots showing summarized frequencies and total numbers of $CD11b^+F4/80^+$ cells in 1g of adipose tissue of adult (~30 weeks old) CTRL and NeoATB mice.
E. Representative FACS plots of $CD4^+CD25^+Foxp3^+$ or $CD4^+CD25^+Foxp3^+ST2^+$ Treg cells in the adipose tissue of CTRL and NeoATB mice. Dot plots showing summarized frequencies and total numbers of $CD4^+CD25^+Foxp3^+$ or $CD4^+CD25^+Foxp3^+ST2^+$ Treg cells in 1g of adipose tissue of CTRL and NeoATB mice.
F. Representative FACS plots showing the frequency of innate lymphoid cells type 2 (ILC2) (gated as: $CD45^+Lin^-SCA1^+ST2^+$) in adipose tissue of adult (~30 weeks old) CTRL and NeoATB mice. Dot plots showing summarized frequencies and total numbers of ILC2 cells in adipose tissue of CTRL and NeoATB mice.

Data are polled from 2-3 independent experiments. Each point in the dot plot figures represents one individual animal. Statistical analysis was performed using Student's t-test (* $p<0.05$; ** $p<0.01$; *** $p<0.001$; **** $p<0.0001$).

Figure S1

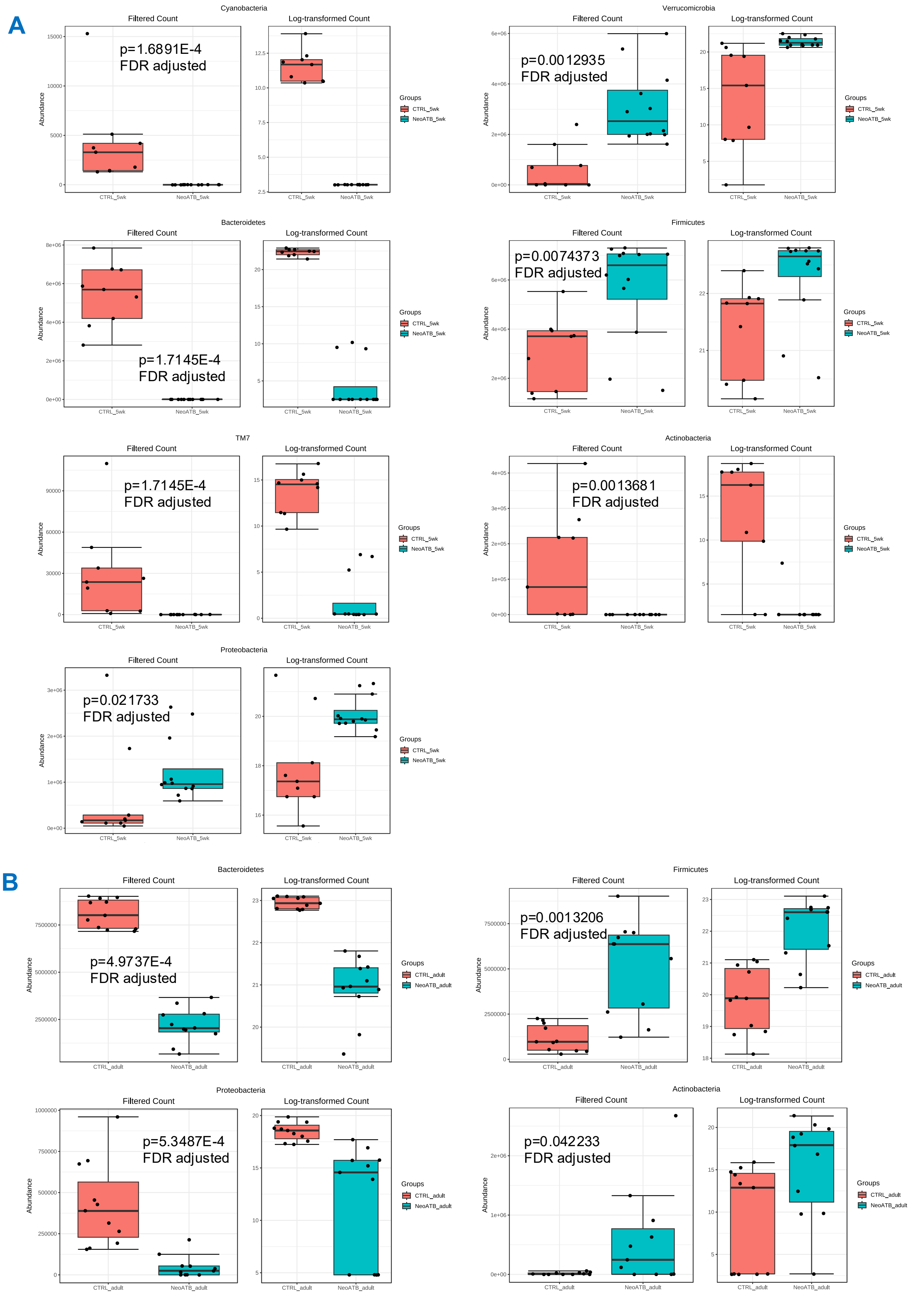
A
Cyanobacteria
Filtered Count
Log-transformed Count
p=1.6891E-4
FDR adjusted
Verrucomicrobia
p=0.0012935
FDR adjusted
Bacteroidetes
p=1.7145E-4
FDR adjusted
Firmicutes
p=0.0074373
FDR adjusted
TM7
p=1.7145E-4
FDR adjusted
Actinobacteria
p=0.0013681
FDR adjusted
Proteobacteria
p=0.021733
FDR adjusted
Abundance
Groups
CTRL_5wk
NeoATB_5wk
B
Bacteroidetes
p=4.9737E-4
FDR adjusted
Firmicutes
p=0.0013206
FDR adjusted
Proteobacteria
p=5.3487E-4
FDR adjusted
Actinobacteria
p=0.042233
FDR adjusted
CTRL_adult
NeoATB_adult

Figure S2

Linear Discriminant Analysis Effect Size (LEfSe)

A. Filtered and Log-transformed counts were used to calculate LEfSe in 5-week-old mice.
B. Filtered and Log-transformed counts were used to calculate LEfSe in adult mice.

Data are pooled from 2-3 independent experiments. Statistical analysis was performed using the Kruskal-Wallis rank sum test, FDR adjusted p=0.05.

Figure S2

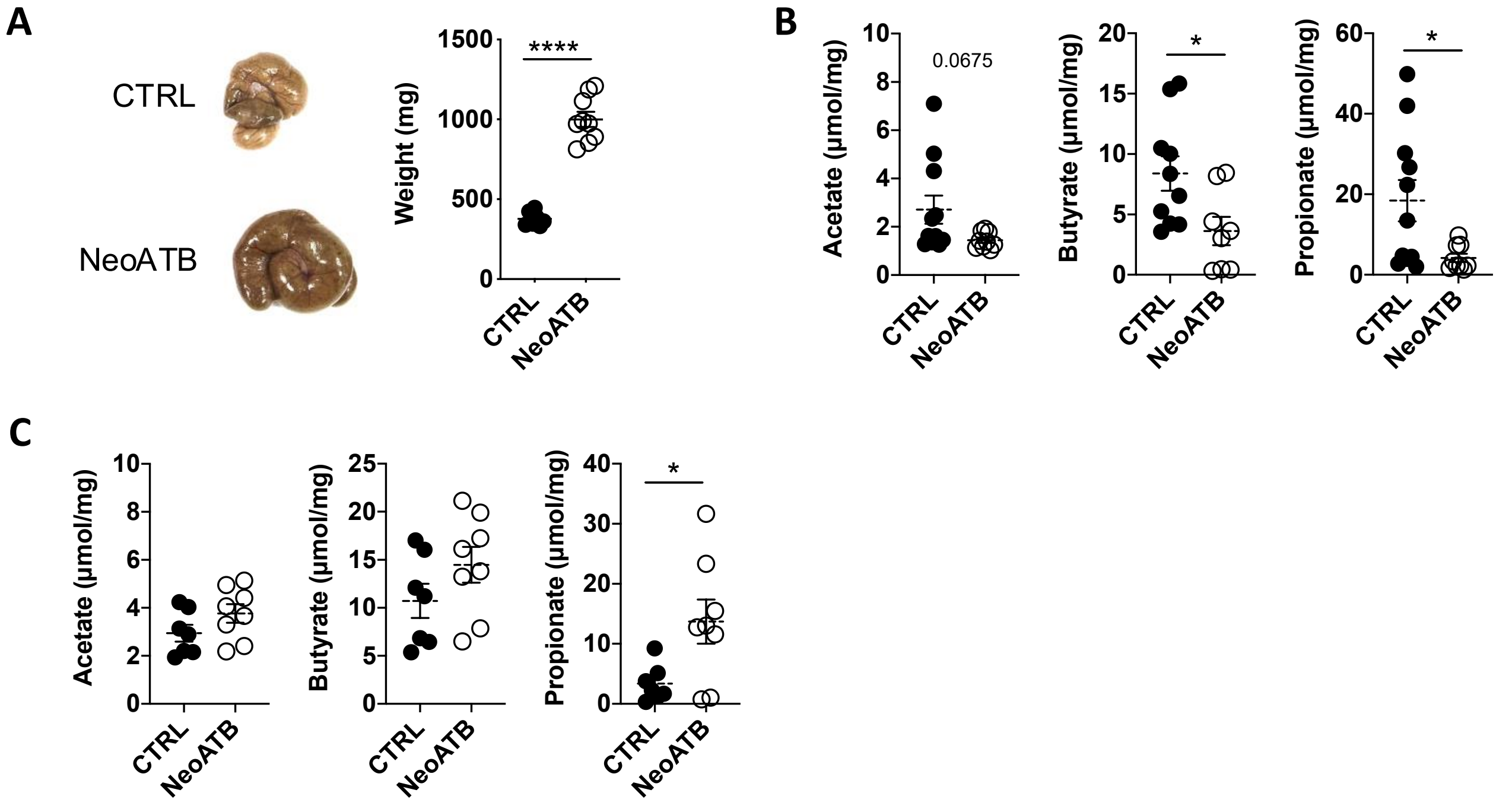

A. Photograph of the cecum of 5 weeks old control and NeoATB mice. The dot plot shows cecum weight (mg) in control and NeoATB mice.
B. Fecal levels of short-chain fatty acids (SCFA) determined by GC-MS in feces of 5-week-old control and NeoATB mice.
C. Fecal levels of short-chain fatty acids (SCFA) determined by GC-MS in feces of 30-week-old control and NeoATB mice.

Data are representative of 2 independent experiments. Statistical analysis was performed using Student's t-test (**** $p<0.0001$).

Figure S3

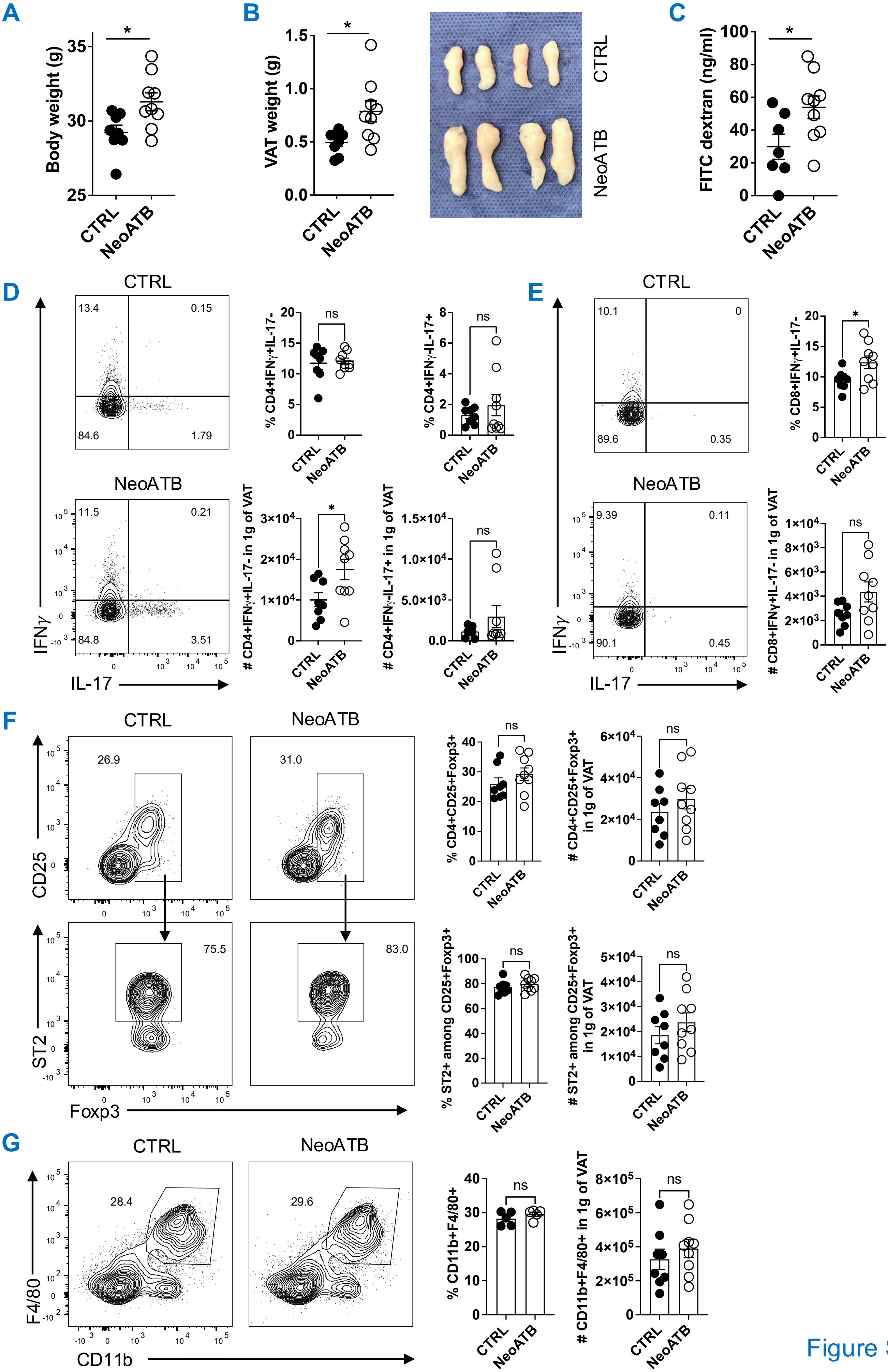
A
Body weight (g)
CTRL
NeoATB
B
VAT weight (g)
CTRL
NeoATB
C
FITC dextran (ng/ml)
D
IFNγ
IL-17
13.4
0.15
84.6
1.79
11.5
0.21
84.8
3.51
% CD4+IFNγ+IL-17-
% CD4+IFNγ-IL-17+
# CD4+IFNγ+IL-17- in 1g of VAT
# CD4+IFNγ-IL-17+ in 1g of VAT
ns
*
E
10.1
0
89.6
0.35
9.39
0.11
90.1
0.45
% CD8+IFNγ+IL-17-
# CD8+IFNγ+IL-17- in 1g of VAT
F
CD25
ST2
Foxp3
26.9
31.0
75.5
83.0
% CD4+CD25+Foxp3+
# CD4+CD25+Foxp3+ in 1g of VAT
% ST2+ among CD25+Foxp3+
# ST2+ among CD25+Foxp3+ in 1g of VAT
G
F4/80
CD11b
28.4
29.6
% CD11b+F4/80+
# CD11b+F4/80+ in 1g of VAT

Figure S4

Fecal microbiota transfer from 5-week-old NeoATB mice can transfer obesity phenotype to germ-free mice.

A. The summarizing dot plot shows the body weight of germ-free mice implanted with feces collected from 5-week-old control (CTRL) or NeoATB mice.

B. The summarizing dot plot shows the weight of white adipose tissue (VAT) in germ-free mice implanted with feces collected from 5-week-old control (CTRL) or NeoATB mice. Photograph of VAT of germ-free mice implanted with feces collected from 5-week-old control (CTRL) or NeoATB mice.

C. Increased intestinal permeability determined by FITC-dextran assay in germ-free mice implanted with feces collected from 5-week-old NeoATB mice.

D. Representative FACS plots show the frequency and total number of CD4+IFNg+IL-17- or CD4+IFNg-IL-17+ cells in the VAT of germ-free mice implanted with feces collected either from 5-week-old control (CTRL) or NeoATB mice.

E. Representative FACS plots show the frequency and total number of CD8+IFNg+IL-17- cells in the VAT of germ-free mice implanted with feces collected from 5-week-old control (CTRL) or NeoATB mice.

F. Representative FACS plots show the frequency and total number of CD4+Foxp3+ or CD4+Foxp3+ST2+ cells in the VAT of germ-free mice implanted with feces collected from 5-week-old control (CTRL) or NeoATB mice.

G. Representative FACS plots show the frequency and total number of macrophages (CD11b+F4/80+) in the VAT of germ-free mice implanted with feces collected from 5-week-old control (CTRL) or NeoATB mice.

Data are representative of 2 independent experiments. Statistical analysis was performed using Student's t-test (* $p<0.05$).

Figure S4

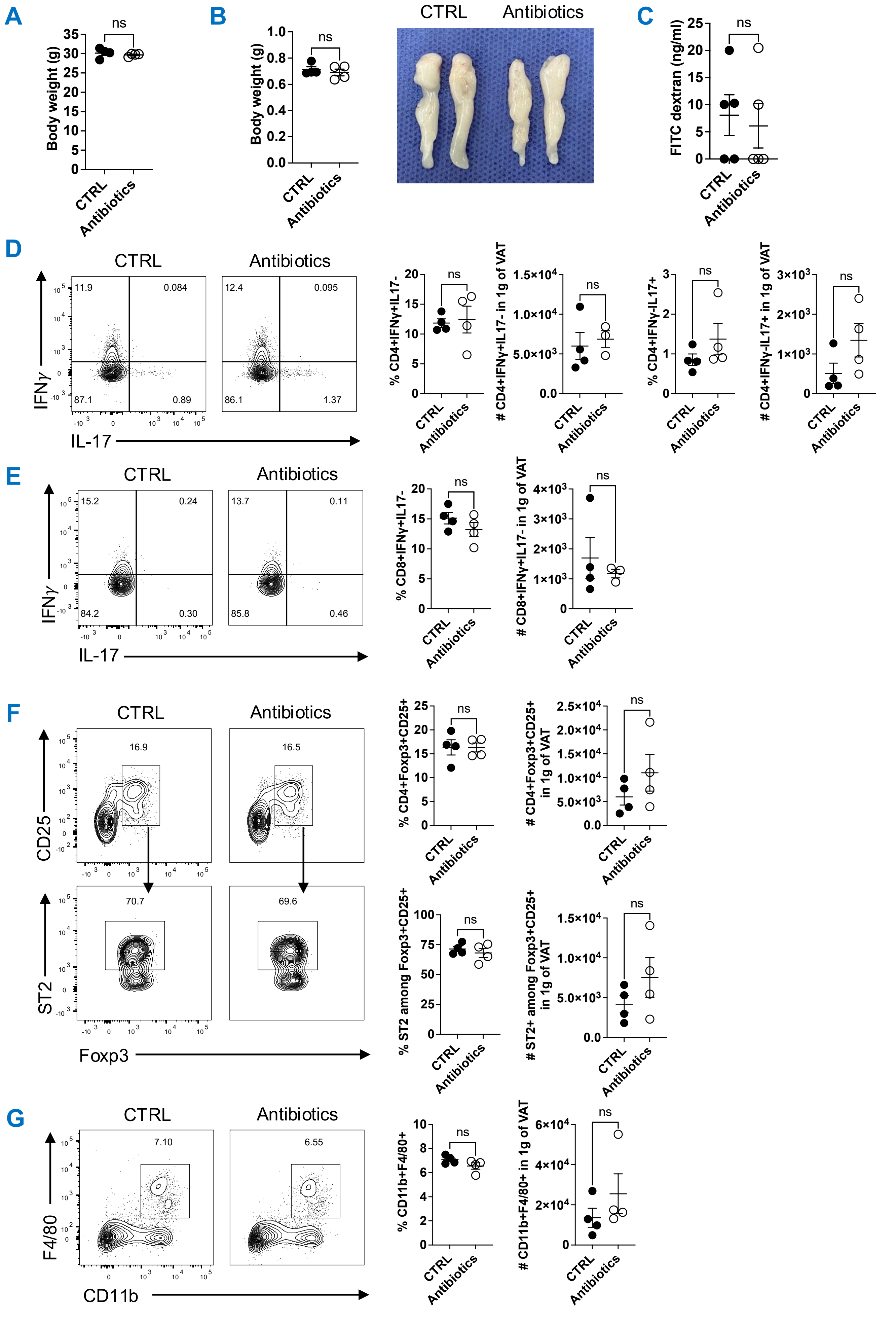

A
B
C
D
E
F
G
ns
Body weight (g)
CTRL
Antibiotics
FITC dextran (ng/ml)
IFNγ
IL-17
Foxp3
CD25
ST2
F4/80
CD11b
% CD4+IFNγ+IL17-
# CD4+IFNγ+IL17- in 1g of VAT
% CD4+IFNγ-IL17+
# CD4+IFNγ-IL17+ in 1g of VAT
% CD8+IFNγ+IL17-
# CD8+IFNγ+IL17- in 1g of VAT
% CD4+Foxp3+CD25+
# CD4+Foxp3+CD25+ in 1g of VAT
% ST2 among Foxp3+CD25+
# ST2+ among Foxp3+CD25+ in 1g of VAT
% CD11b+F4/80+
# CD11b+F4/80+ in 1g of VAT

Figure S5

Antibiotic treatment per se is insufficient to mimic the obesity phenotype observed in NeoATB mice.

A. The summarizing dot plot shows the body weight of germ-free mice treated with antibiotics or left untreated (CTRL).

B. The summarizing dot plot shows the weight of white adipose tissue (VAT) in germ-free mice treated with antibiotics or left untreated (CTRL).

C. Intestinal permeability was determined by FITC-dextran assay in germ-free mice treated with antibiotics or untreated CTRL mice.

D. Representative FACS plots show the frequency and total number of CD4+IFNg+IL-17- or CD4+IFNg-IL-17+ cells in the VAT of germ-free mice treated with antibiotics or left untreated (CTRL).

E. Representative FACS plots show the frequency and total number of CD8+IFNg+IL-17- cells in the VAT of germ-free mice treated with antibiotics or left untreated (CTRL).

F. Representative FACS plots show the frequency and total number of CD4+Foxp3+ or CD4+Foxp3+ST2+ cells in the VAT of germ-free mice treated with antibiotics or left untreated (CTRL).

G. Representative FACS plots show the frequency and total number of macrophages (CD11b+F4/80+) in the VAT of germ-free mice treated with antibiotics or left untreated (CTRL).

Data is representative of 1 experiment. Statistical analysis was performed using Student's t-test (ns = not significant).

Figure S5

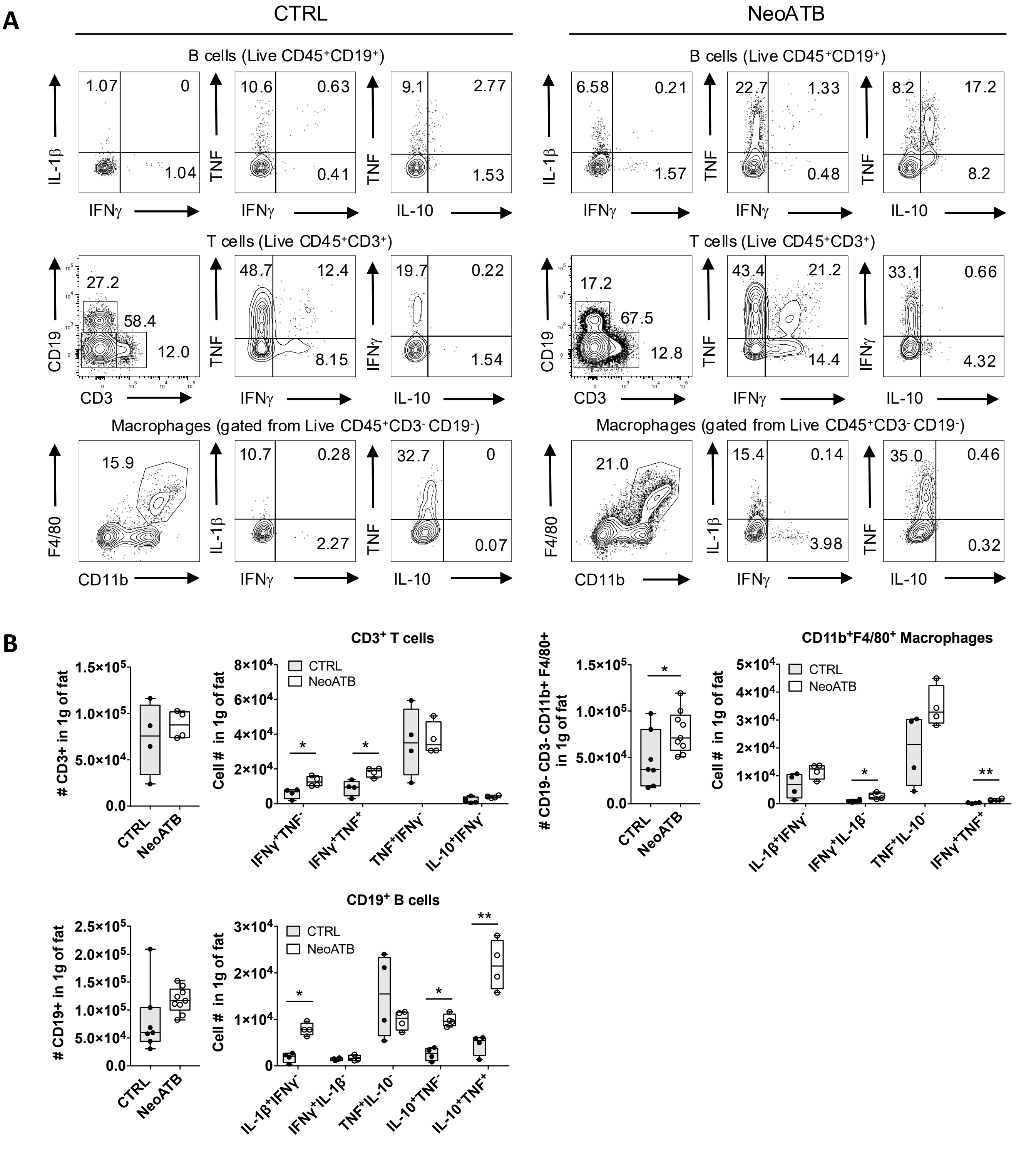

Obesity prone phenotype in young NeoATB mice

A. Representative FACS plots showing frequency of B cells (gated as: Live CD45⁺CD19⁺), total T cells (gated as: Live CD45⁺CD3⁺) and macrophages (gated as: Live CD45⁺CD3⁻CD19⁻ CD11b⁺F4/80⁺) and cytokines (IL-β⁺, IFNγ⁺, TNF⁺ and IL-10⁺) in adipose tissue of 5 weeks old control (CTRL) and NeoATB mice.
B. Summarizing figures showing total number of B cells, T cells, Macrophages and cytokines in adipose tissue of 5 weeks old CTRL and NeoATB mice.

Data are polled of 2 independent experiments. Each point in figures represents one individual animal. Statistical analysis was performed using Student's t-test (* $p<0.05$; ** $p<0.01$).

Figure S6

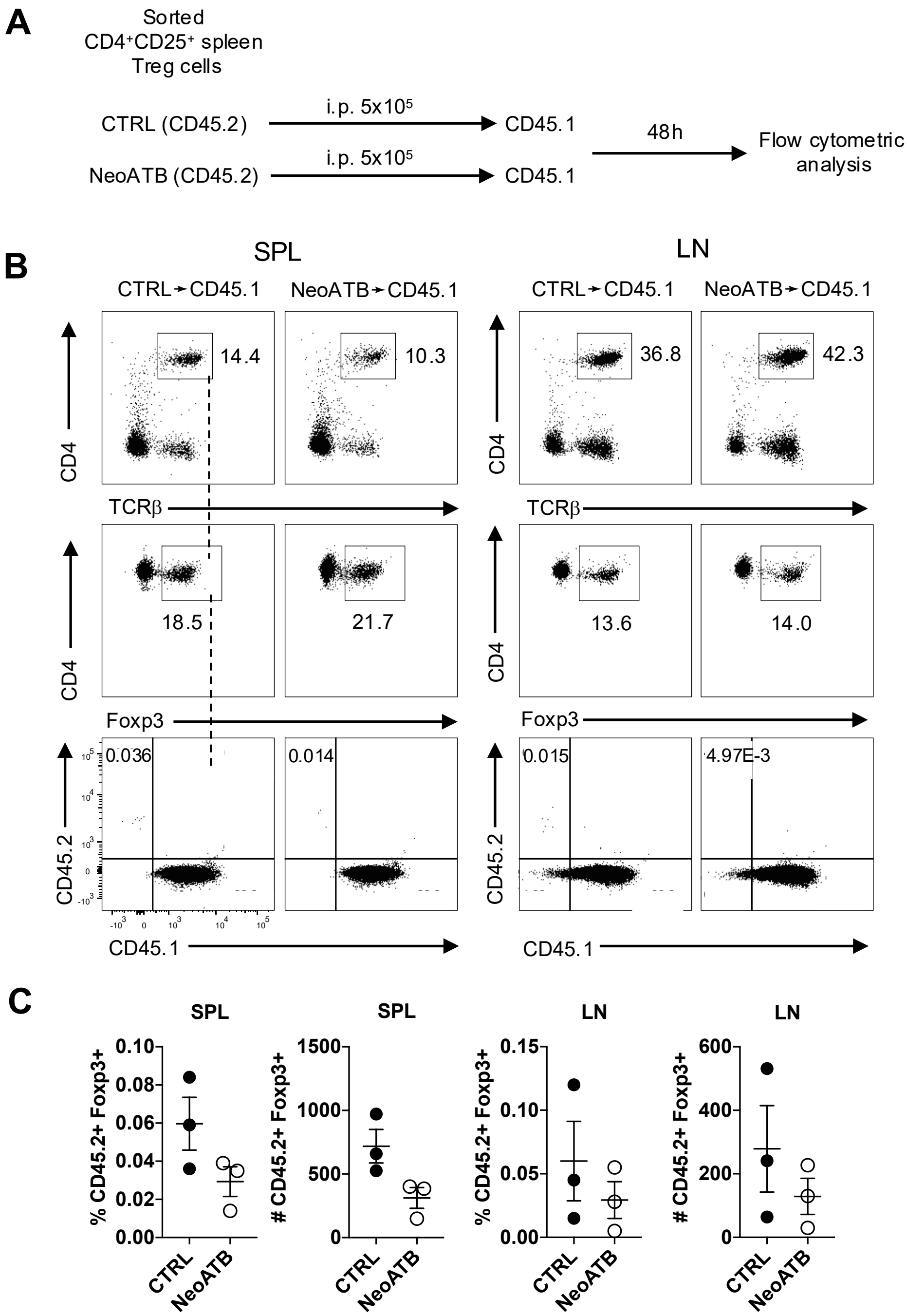

Reduced migration of NeoATB Treg cells.

A. Scheme of experiment design. Splenic Treg cells were sorted from age matched control (CTRL, CD45.2+) and NeoATB mice (CD45.2+). Sorted Treg cells ($5x10^5$) were i.p. injected into syngeneic CD45.1+ mice. Migration of Treg cells was examined in the spleens (SPL) and peripheral lymph nodes (LN) 48h after i.p. injections.
B. Representative FACS plots showing gating strategy as well as frequency of CD4+, Foxp3+ and CD45.1+ vs CD45.2+ cells in indicated tissues.
C. Dot plots showing frequencies and total number of CD45.2+ CTRL and NeoATB Treg cells in indicated tissues.

Data are polled of 2 independent experiments. Each point in figures represents one individual animal. Statistical analysis was performed using Student's t-test (* $p<0.05$).

Figure S7

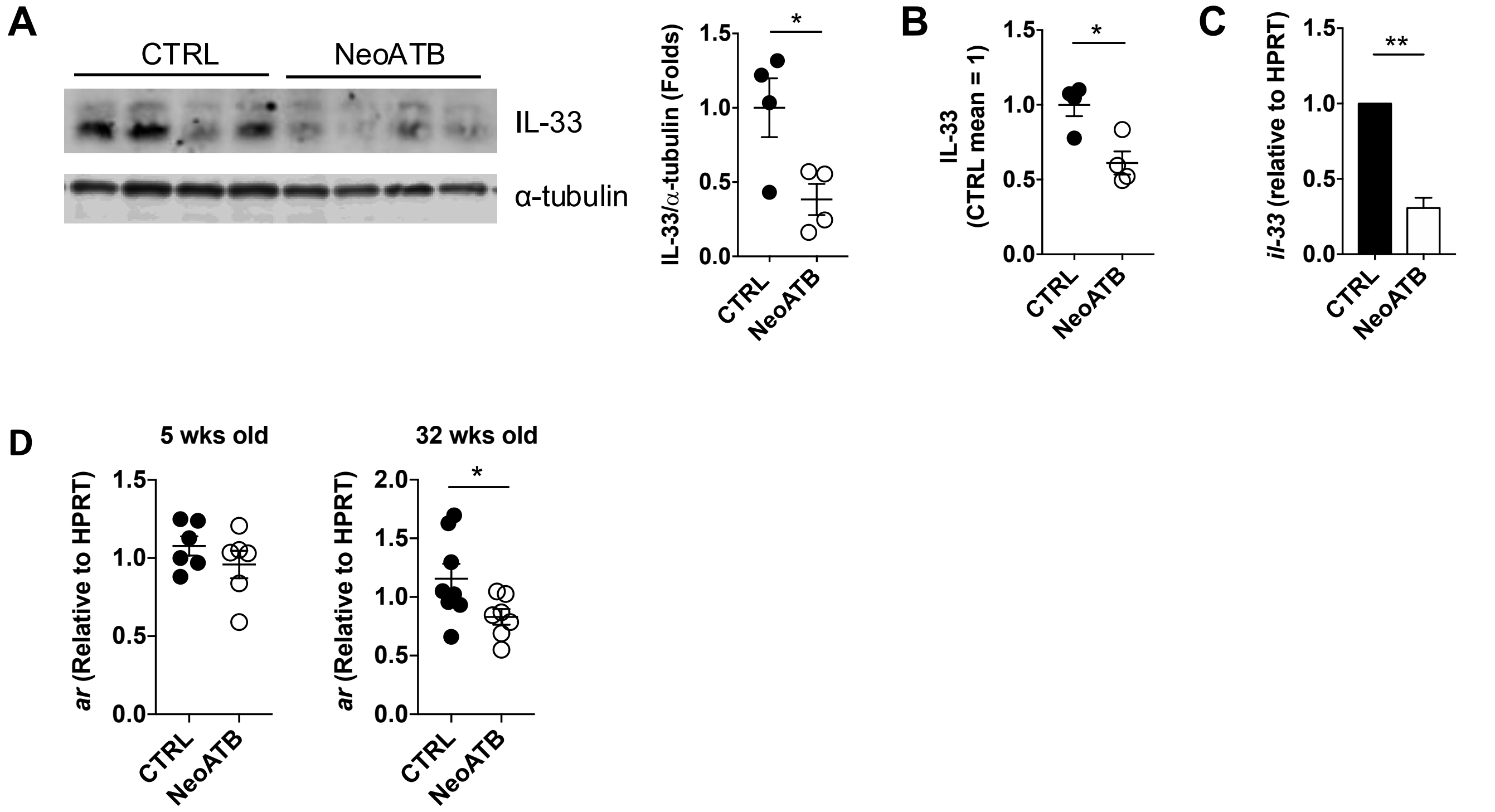

Decrease of IL-33 protein in the fat of young NeoATB mice

A. Western blot analysis of IL-33 protein in the adipose tissue of CTRL and NeoATB mice. The dot plot shows the fold difference of IL-33, determined as a ratio of IL-33 to α-tubulin, in the adipose tissue of CTRL and NeoATB mice.
B. Levels of adipose IL-33 were determined by ELISA and normalized to CTRL levels.
C. Gene expression of interleukin 33 (IL-33) in sorted $CD45^-$ adipocytes of 5-week-old CTRL and NeoATB mice.
D. Gene expression of androgen receptor (*Ar*) in the adipose tissue of 5—and 32-week-old CTRL and NeoATB mice.

Data are polled from 2 independent experiments (C, D) or representative of one experiment (A), or representative of one out of two experiments (B). Each point in the figures represents one individual animal. Statistical analysis was performed using Student's t-test (* $p<0.05$; ** $p<0.01$).

Figure S8

Name	Gene	Catalog number	Company
Interferon gamma	*Ifng*	Mm01168134_m1	Thermo Fisher Scientific
Interleukin 1 beta	*Il1b*	Mm00434228_m1	Thermo Fisher Scientific
Interleukin 4	*Il4*	Mm00445259_m1	Thermo Fisher Scientific
Interleukin 5	*il5*	Mm00439646_m1	Thermo Fisher Scientific
Interleukin 6	*Il6*	Mm00446190_m1	Thermo Fisher Scientific
Interleukin 10	*Il-10*	Mm01288386_m1	Thermo Fisher Scientific
Interleukin 12	*Il12*	Mm01288989_m1	Thermo Fisher Scientific
Interleukin 13	*il13*	Mm00434204_m1	Thermo Fisher Scientific
Transforming growth factor, beta 1	*Tgfb1*	Mm01178820_m1	Thermo Fisher Scientific
Tumor necrosis factor	*Tnf*	Mm00443258_m1	Thermo Fisher Scientific
TGFb receptor I	*Tgfbr1*	Mm_00436964_m1	Thermo Fisher Scientific
TGFb receptor II	*Tgfbr2*	Mm_00436976_m1	Thermo Fisher Scientific
Interleukin 1 receptor-like 1 (ST2)	*Il1rl1*	Mm00516117_m1	Thermo Fisher Scientific
Toll-like receptor 2	*tlr2*	Mm00442346_m1	Thermo Fisher Scientific
Toll-like receptor 4	*tlr4*	Mm00445273_m1	Thermo Fisher Scientific
Occludin	*ocln*	Mm00500910_m1	Thermo Fisher Scientific
Regenerating islet-derived 3 gamma	*reg3g*	Mm00441127_m1	Thermo Fisher Scientific
Regenerating islet-derived 3 beta	*reg3b*	Mm00440616_g1	Thermo Fisher Scientific
Hypoxanthine guanine phosphoribosyl transferase	*Hprt*	Mm00446968_m1	Thermo Fisher Scientific

Supplementary Table 1. TaqMan Assays used for qPCR
